# The Epidemiology of Hepatitis C Virus in the Fertile Crescent: Systematic Review and Meta-Analysis

**DOI:** 10.1371/journal.pone.0135281

**Published:** 2015-08-21

**Authors:** Hiam Chemaitelly, Karima Chaabna, Laith J. Abu-Raddad

**Affiliations:** 1 Infectious Disease Epidemiology Group, Weill Cornell Medical College in Qatar, Cornell University, Qatar Foundation, Education City, Doha, Qatar; 2 Department of Healthcare Policy & Research, Weill Cornell Medical College, Cornell University, New York, New York, United States of America; National Institute of Health, ITALY

## Abstract

**Objective:**

To characterize hepatitis C virus (HCV) epidemiology in countries of the Fertile Crescent region of the Middle East and North Africa (MENA), namely Iraq, Jordan, Lebanon, Palestine, and Syria.

**Methods:**

We systematically reviewed and synthesized available records of HCV incidence and prevalence following PRISMA guidelines. Meta-analyses were implemented using a DerSimonian-Laird random effects model with inverse weighting to estimate the country-specific HCV prevalence among the various at risk population groups.

**Results:**

We identified eight HCV incidence and 240 HCV prevalence measures in the Fertile Crescent. HCV sero-conversion risk among hemodialysis patients was 9.2% in Jordan and 40.3% in Iraq, and ranged between 0% and 3.5% among other populations in Iraq over different follow-up times. Our meta-analyses estimated HCV prevalence among the general population at 0.2% in Iraq (range: 0–7.2%; 95% CI: 0.1–0.3%), 0.3% in Jordan (range: 0–2.0%; 95% CI: 0.1–0.5%), 0.2% in Lebanon (range: 0–3.4%; 95% CI: 0.1–0.3%), 0.2% in Palestine (range: 0–9.0%; 95% CI: 0.2–0.3%), and 0.4% in Syria (range: 0.3–0.9%; 95% CI: 0.4–0.5%). Among populations at high risk, HCV prevalence was estimated at 19.5% in Iraq (range: 0–67.3%; 95% CI: 14.9–24.5%), 37.0% in Jordan (range: 21–59.5%; 95% CI: 29.3–45.0%), 14.5% in Lebanon (range: 0–52.8%; 95% CI: 5.6–26.5%), and 47.4% in Syria (range: 21.0–75.0%; 95% CI: 32.5–62.5%). Genotypes 4 and 1 appear to be the dominant circulating strains.

**Conclusions:**

HCV prevalence in the population at large appears to be below 1%, lower than that in other MENA sub-regions, and tending towards the lower end of the global range. However, there is evidence for ongoing HCV transmission within medical facilities and among people who inject drugs (PWID). Migration dynamics appear to have played a role in determining the circulating genotypes. HCV prevention efforts should be targeted, and focus on infection control in clinical settings and harm reduction among PWID.

## Introduction

Hepatitis C virus (HCV) is a main risk factor for liver cancer and cirrhosis and is estimated to affect 1–3% of the population in most countries globally [1, 2]. The vast majority of HCV transmission is blood-borne and largely preventable [3, 4]. Recent breakthroughs in HCV treatment research suggest promising prospects for treating and containing the infection, and raise hope for an improved quality of life among infected individuals [5].

In few countries of the Middle East and North Africa (MENA) region, namely Egypt and Pakistan, HCV is prevalent at high levels: 14.7% in Egypt [6, 7] and 4.8% in Pakistan [8, 9]. Meanwhile, infection levels remain poorly estimated for most MENA countries including those in the Fertile Crescent (FC). Geographically, for this study, we define the FC subregion of MENA to include Iraq, Jordan, Lebanon, Palestine, and Syria. Since Egypt was covered by a separate systematic review published recently [7], we did not include Egypt as part of our study. FC countries share historical, socio-cultural, and geo-political similarities, and their health care systems are often directly or indirectly interlinked or affected by decades of political turmoil and refugee waves within this territory.

Our study primarily aims at characterizing HCV epidemiology in these countries by 1) systematically reviewing and synthesizing all available published and unpublished records of HCV incidence and prevalence among the different population groups, and 2) estimating the population-level country-specific HCV prevalence by pooling available HCV prevalence measures among the general population in each country. A secondary outcome of our study is an analysis of HCV genotype diversity. This work is conducted under the umbrella of the MENA HCV Epidemiology Synthesis Project; an ongoing effort to characterize HCV epidemiology and inform key public health research, policy, and programming priorities in MENA [7, 10–15].

## Materials and Methods

### Data sources and search strategy

We systematically reviewed all HCV incidence (Table 1) and prevalence (Tables 2 and 3 and S2 Table) data in FC following the Preferred Reporting Items for Systematic Reviews and Meta-analyses (PRISMA) guidelines [16]. The PRISMA checklist can be found in S1 Fig. The methodology was adapted from a recently published protocol for a systematic review of HCV incidence and prevalence measures for countries in the Horn of Africa subregion of MENA [10, 17]. Our search criteria can be found in S2 Fig. Briefly, we surveyed PubMed and Embase (up to April 23, 2015), different regional databases (up to November 5–16, 2014), the abstract archives of the International AIDS Society conferences (up to November 16, 2014), and country-level and international organizations’ reports available through the MENA HIV/AIDS Epidemiology Synthesis Project database (up to April 23, 2015) [18, 19]. We used a broad search criteria with no language restrictions. PubMed and Embase were searched using text terms and MeSH/Emtree terms exploded to cover all subheadings. Our search was restricted to articles published after 1989, the year when HCV was first discovered [20, 21].

**Table 1:** Studies reporting hepatitis C virus (HCV) incidence in countries of the Fertile Crescent.

First author, year of publication [citation]	Year of data collection	Study site	Population’s classification based on risk of HCV exposure	Population	Sample size at recruitment	Lost to follow-up	HCV sero-conversion risk (relative to total sample size)	Duration of follow-up
**Jordan**								
Batieha, 07 [39]	2003	Dialysis units/National	High risk population	Hemodialysis patients	1300		9.2%	12 months
**Iraq**								
Al-Rubaie, 11 [40]	2009	Hospital	High risk population	Hemodialysis patients	57	0	40.3%	12 months
Al-Jadiry, 08 [42]	2007	Hospital	Special clinical population	Pediatric patients with acute lymphoblastic leukemia	123	0	3.2%	30 months (median)
Al-Kubaisy, 00 [44]		Hospital	High risk population	Newborns to HCV infected women	26	0	0%	6 months
Al-Ali, 14 [41]	2006–07	Hospital	Special clinical population	Pediatric cancer patients on chemotherapy	85	22	3.2%	12 months
Al-Ani, 11 [43]	2007–09	Hospital	Low risk population	Healthy children	60	0	0%	6 months
Al-Ani, 11 [43]	2007–09	Hospital	Special clinical population	Pediatric patients with leukemia on chemotherapy	29	0	3.5%	6 months
Al-Ani, 11 [43]	2007–09	Hospital	Special clinical population	Pediatric patients with leukemia who have had their baseline screening prior to chemotherapy	27	0	0%	6 months

**Table 2:** Studies reporting hepatitis C virus (HCV) prevalence among populations at high risk in countries of the Fertile Crescent.

First author, year of publication [citation]	Years of data collection		Study site	Study sampling procedure	Population	Sample size*	HCV prev**
**Iraq (n = 36)**							
Abdul-Aziz, 01 [46]	1999–01		Central laboratory	Convenience	Hemodialysis patients	95	0%
Abdul-Aziz, 01 [46]	1999–01		Central laboratory	Convenience	Thalassemic patients	163	8%
Abdul-Karim, 11 [149]	2008		Hospital	Convenience	Patients with bleeding disorders	243	40.3%
Abdullah, 12 [48]	2010		Hemodialysis centers	Convenience	Hemodialysis patients	236	39%
Abdullah, 12 [47]	2005–07		Dialysis unit	Convenience	Hemodialysis patients	80	28.7%
Abed, 10 [150]	2008		Thalassemia center	Convenience	Thalassemic patients	111	46%
Albahadle, 13 [151]	2011		Thalassemia center	Convenience	Thalassemic patients (0–18 years)	206	19.9%
Al-Barzinji, 06 [152]	2004		Hospital	Convenience	Multi-transfused leukemia patients on chemotherapy (1–12 years)	88	4.5%
Al-Beldawi, 10 [56]	2006		Hemophilia center	Convenience	Hemophilia patients (<20 years)	200	40%
Al-Dulaimi, 12 [49]	2010–11		Hospital	Convenience	Hemodialysis patients	84	14.3%
Al-Greti, 13 [153]	2011–12		Thalassemia center	Convenience	Thalassemic patients	100	37%
Al-Juboori, 12 [154]	2010–11		Thalassemia center	Convenience	Thalassemic patients (<20 years)	50	10%
Al Kubaisy, 06 [45]	1998		Hospital	SRS	Thalassemic patients (2–10 years)	559	67.3%
Al-Marzoqi, 09 [66]	2008		Thalassemia center	Convenience	Thalassemic patients (children)	50	38%
Al-Mashhadani, 07 [50]	2002		Hospital	Convenience	Hemodialysis patients	87	11.5%
Al-Thwani, 06 [57]	1997–04		Blood trans center	Convenience	Hemophilia patients	100	25%
Al Wtaify, 00 [155]	1998–99		Thalassemia center	Convenience	Thalassemic patients (children)	200	9.5%
Al-Zamili, 09 [156]	2007–08		Hospital	Convenience	Thalassemic patients (<20 years)	325	4%
Easa, 09 [157]	2007–08		Thalassemia center	Convenience	Thalassemic patients (5–18 years)	140	26.4%
Fadhil, 12 [158]	2010–11		Thalassemia center	Convenience	Thalassemic patients	200	21%
Hashem, 13 [159]	2011		Hereditary blood diseases center	Convenience	Thalassemic patients	284	19%
Hassan, 08 [137]	1996–01		Central laboratory	Convenience	Thalassemic patients	136	16.9%
Khaled, 14 [160]	2012		Thalassemia center	Convenience	Thalassemic patients	480	10.4%
Khalid, 12 [161]			Thalassemia center	Convenience	Thalassemic patients	200	17.5%
Khattab, 08 [51]	2003–05		Renal transplant center	Convenience	Hemodialysis patients	169	7.1%
Khattab, 10 [52]	2003–08		Renal transplant center	Convenience	Hemodialysis patients	244	4.9%
Mnuti, 11 [53]	2008–10		Hemodialysis unit	Convenience	Hemodialysis patients	100	41%
Muhsin, 13 [58]	2011–12		Hospital	SRS	Hemophilia patients	60	6.7%
Muhsin, 13 [58]	2011–12		Hospital	SRS	Thalassemic patients	56	25%
Mustafa, 10 [162]	2003–04		Thalassemia center	Convenience	Thalassemic patients (<20 years)	626	28.1%
Omer, 11 [163]	2006–08		Hospital	Convenience	Multi-transfused leukemia patients	291	3.4%
Omer, 11 [164]	2010		Thalassemia center	Convenience	Thalassemic patients	54	7.4%
Raham, 11 [165]	1999–00		Thalassemia center	Convenience	Thalassemic patients	110	26.4%
Ramzi, 10 [54]	2009		Dialysis center	Convenience	Hemodialysis patients	101	26.7%
Saadoon, 12 [64]			Blood trans center	Convenience	Thalassemic patients	162	34.6%
Shihab, 14 [55]	2012–13		Hospital	Convenience	Hemodialysis patients	122	42.6%
**Jordan (n = 7)**							
Al-Jamal, 09 [166]	2007–08		Hospitals	Convenience	Hemodialysis patients	120	28%
Al-Sweedan, 11 [68]	2008		Thalassemia unit	Convenience	Thalassemic patients	122	32.8%
Batchoun, 11 [167]			Hospitals	Convenience	Hemodialysis patients	134	47.7%
Batieha, 07 [39]	2003		Hemodialysis units	Convenience	Hemodialysis patients	1711	21%
Bdour, 02 [111]			Hemodialysis units	Convenience	Hemodialysis patients	283	32.5%
Ghunaimat, 07 [168]			Hemodialysis unit	Convenience	Hemodialysis patients	209	49.8%
Said, 95 [169]	1994		Hemodialysis centers	Convenience	Hemodialysis patients	273	24.5%
**Lebanon (n = 6)**							
Abdelnour, 97 [72]			Hospitals	Convenience	Hemodialysis patients	108	16%
Inati, 09 [74]			Thalassemia center	SRS	Thalassemic patients	200	0%
Mahfoud, 10 [71]	2007–08		Not applicable	RDS	People who inject drugs	106	52.8%
Naman, 96 [73]			Hemodialysis centers	Convenience	Hemodialysis patients	317	27%
Ramia, 02 [75]	1999–00		Thalassemia center	Convenience	Thalassemic patients	395	14.0%
Ramia, 03 [76]			Hospital	Convenience	Multi-transfused cancer patients	65	4.6%
**Palestine (n = 3)**							
Dumaidi, 14 [87]		2012–13	Hemodialysis units	Convenience	Hemodialysis patients	146	27.4%
El-Kader, 10 [88]		2007	Hospital	Convenience	Hemodialysis patients	246	17.9%
Stulhofer, 12 [89]		2010	Different localities	RDS	People who inject drugs	199	45.2%
**Syria (n = 5)**							
Abdulkarim, 98 [92]			Dialysis unit	Convenience	Hemodialysis patients	120	75%
Ali, 12 [96]		2007–11	Hospital	Convenience	Hemophilia patients	375	20.5%
Syrian MOH, 08 [95]		2006	Not applicable	Snowball	Drug users including people who inject drugs	336	21%
Moukeh, 09 [93]		2006	Hospital	Convenience	Hemodialysis patients	550	54.4%
Othman, 01 [170]		1996	Hospital	Convenience	Hemodialysis patients	139	48.9%

MENA HIV/AIDS ESP, Middle East and North Africa HIV/AIDS Epidemiology Synthesis Project database; MOH, Ministry of Health; Prev, prevalence; RDS, respondent driven sampling; SRS, simple random sampling; Trans, transfusion.

*The table reports only studies whose sample size is greater or equal to 50 participants. For space considerations, the table shows the overall HCV measure of each study rather than stratifications within population subgroups.

**The decimal places of the prevalence figures are as reported in the original report, but prevalence figures with more than one decimal places were rounded to one decimal place.

**Table 3:** Studies reporting hepatitis C virus (HCV) prevalence among the general population (populations at low risk) in countries of the Fertile Crescent.

First author, year of publication [citation]	Years of data collection	Study site	Study sampling procedure	Population	Sample size*	HCV prev**
**Iraq (n = 45)**						
Abdul-Aziz, 01 [46]	1999–01	Central laboratory	Convenience	Blood donors	20108	0.8%
Abdul-Aziz, 01 [46]	1999–01	Central laboratory	Convenience	New employees	257	0.4%
Abdullah, 12 [47]	2005–07		Convenience	Blood donors	100	1%
Al-Ani, 11 [43]	2007–09	Hospital	Convenience	Children	60	0%
Al-Azzawi, 06 [171]	2004	Primary health centers	Convenience	Pregnant women	100	1%
Al-Doori, 06 [172]	2004–05	Central laboratory	Convenience	Blood donors	1978	0.4%
Al-Duliami, 12 [173]	2010–11	Central laboratory	SRS	Blood donors	90	0%
Al-Greti, 13 [153]	2011–12		Convenience	General population	50	4%
Al-Hamdani, 12 [174]	2003–04, 06, 08–09	Hospital	Convenience	New employees	4162	2.6%
Ali, 09 [175]	2007–08	Blood bank	Convenience	Blood donors	600	1.7%
Al-Jebori, 10 [176]	2003–09	Central laboratory	Convenience	Mixed general population	120460	0.2%
Aljooani, 12 [177]	2005–07	Hospital	Convenience	Blood donors	430	2.8%
Al-Juboury, 10 [178]	2007–08	Blood bank	Convenience	Blood donors	23336	0.5%
Al-Kamil, 11 [179]	2006–08	Central blood bank	Convenience	Blood donors	161987	0.1%
Al-Kubaisy, 02 [102]		Hospitals & health care centers	SRS	Pregnant women	3491	3.2%
Al-Saad, 09 [61]	2008	Hospital	Convenience	General population	100	0%
Al Wtaify, 00 [155]	1998–99	Hospital	SRS	Outpatient hospital attendees (children)	200	0.5%
Al-Zamili, 09 [156]	2007–08	Hospital	Convenience	Children	325	0%
Amin, 12 [180]	2008–09	Blood bank	Convenience	Blood donors	35540	0.1%
Ataallah, 11 [181]	2006–09	National blood trans center	Convenience	Blood donors	495648	0.3%
Chiad, 09 [182]	2003–04	National blood trans center	Convenience	Blood donors	1000	1%
Chironna, 03 [105]		Refugee camp	Convenience	Refugees	637	0.1%
Fadhil, 12 [158]	2010–11	Hospital	Convenience	Outpatient hospital attendees	50	0%
Fawzi, 11 [183]	2010	Hospital & health centers	Convenience	Pregnant women	520	1.1%
Hamim, 12 [184]	2006–10	Main blood trans center	Convenience	Outpatient hospital attendees	4886	0.9%
Hamim, 12 [185]	2008–09	Blood trans centers	Convenience	Blood donors	871973	0.4%
Hassan, 08 [137]	1996–01	Central laboratory	Convenience	Blood donors	42140	0.1%
Hussain, 08 [186]	2004–05	Hospital	Convenience	General population	108	0%
Hussein, 10 [187]	2006–07	Central blood bank	Convenience	Blood donors	31574	0.1%
Hussain, 10 [188]	2008–09		Convenience	Blood donors	117	0.8%
Jassim, 11 [189]			Convenience	Blood donors	170	0%
Khalid, 12 [161]		Central blood bank	Convenience	Blood donors	100	2%
Naji, 13 [190]	2011–12	Central laboratory	Convenience	Newly married individuals	200	1.5%
Noaman, 12 [63]	2009–10	Blood bank	Convenience	Blood donors	93	1.1%
Obied, 14 [100]	2012–13	Central blood bank	Convenience	Blood donors	5179	0.4%
Omer, 11 [163]	2006–08	Hospital	Convenience	General population	350	0.3%
Richter, 14 [191]	2011	Community	Convenience	First generation immigrants	290	0.3%
Saadoon, 08 [192]	2007	Hospitals	Convenience	Pregnant women	875	5.1%
Saadoon, 12 [64]		Blood bank	Convenience	Blood donors	1128	0.6%
Salih, 07 [193]	2007	Hospital	Convenience	Blood donors	95	2.1%
Salman, 07 [194]	2006–07	Antenatal clinics	Convenience	Pregnant women	60	0%
Tarky, 13 [59]	2005–06	Households	StRS	General population	9610	0.4%
Tawfeeq, 13 [101]	2011–12	Blood bank	Convenience	Blood donors	15560	0.3%
Toffik, 06 [195]		Hospital	Convenience	Outpatient clinic attendees	100	0%
Toffik, 06 [195]			Convenience	Blood donors	200	0.5%
**Jordan (n = 5)**						
Al Abbadi, 14 [196]	2009–11	Blood bank	Convenience	Blood donors	94270	0.1%
Al-Gani, 11 [197]	2006–09	Blood bank	Convenience	Blood donors	8190	0.9%
Hamoudi, 13 [112]		Health centers	SRS	Health centers’ attendees	706	0.4%
Jbara, 06 [198]	2003–05	Blood bank	Convenience	Blood donors	8750	0.8%
Rashdan, 08 [103]	2004–06	Blood bank	Convenience	Blood donors	14236	0.2%
**Lebanon (n = 13)**						
Araj, 95 [77]			SRS	Blood donors	4179	0.1%
Baddoura, 02 [84]		Laboratories (nation-wide)	Convenience	General population (Lebanese)	2879	0.6%
Baddoura, 02 [84]		Laboratories (nation-wide)	Convenience	General population (immigrants)	103	2.9%
Irani-Hakime, 01 [199]	1999	Blood bank	Convenience	Blood donors	600	0.2%
Irani-Hakime, 06 [78]	1997–03	Blood bank	Convenience	Blood donors	16084	0.4%
Nabulsi, 97 [80]	1993–95	Antenatal clinics		Pregnant women	558	0%
Naman, 96 [73]				Blood donors	7771	0.4%
Ramia, 03 [76]		Hospital	SRS	Blood donors	500	0.2%
Ramia, 05 [81]	2002–03	Blood bank	SRS	Blood donors	56	0%
Salem, 03 [82]		Hospital	SRS	Outpatient hospital attendees	70	0%
Salem, 03 [82]		Hospital	SRS	Blood donors	150	0%
Tamim, 01 [83]	1998–00	Blood bank	Convenience	Blood donors (Lebanese)	5027	0.3%
Tamim, 01 [83]	1998–00	Blood bank	Convenience	Blood donors (non-Lebanese)	88	3.4%
**Palestine (n = 11)**						
PHIC, 14 [33]	2013	Blood banks (hospitals)		Blood donors	19571	0.2%
PHIC, 14 [33]	2013	National blood bank		Blood donors	9643	0.3%
MENA HIV ESP, 11 [18, 32]	2011			Blood donors	63477	0.2%
PHIC, 10 [34]	2009	Blood banks (hospitals)		Blood donors	48001	0.2%
PHIC, 08 [35]	2007	Blood banks (hospitals)		Blood donors	49105	0.3%
PHIC, 07 [36]	2006			Blood donors	53151	0.2%
PHIC, 06 [37]	2005	Blood bank		Blood donors	45029	0.2%
PHIC, 04 [38]	2003	Blood bank		Blood donors	44990	0.3%
Novack, 07 [91]	1999–02	Blood bank	Convenience	Blood donors	2784	3.9%
Shemer-Avni, 98 [90]		Blood bank	Convenience	Blood donors	1509	2.2%
Shemer-Avni, 98 [90]		Hospital	SRS	Outpatient hospital attendees	124	9%
**Syria (n = 10)**						
Ali, 10 [200]	2000–07	Blood trans center	Convenience	Blood donors	131403	0.4%
MENA HIV ESP, 10 [18, 32]	Q 1, 2006			Blood donors	55549	0.3%
MENA HIV ESP, 10 [18, 32]	Q 2, 2006			Blood donors	87920	0.4%
MENA HIV ESP, 10 [18, 32]	Q 3, 2006			Blood donors	77734	0.6%
MENA HIV ESP, 10 [18, 32]	Q 4, 2006			Blood donors	83347	0.5%
MENA HIV ESP, 10 [18, 32]	Q 1, 2007			Blood donors	83501	0.4%
MENA HIV ESP, 10 [18, 32]	Q 2, 2007			Blood donors	92452	0.4%
MENA HIV ESP, 10 [18, 32]	Q 3, 2007			Blood donors	83560	0.5%
MENA HIV ESP, 10 [18, 32]	2011			Blood donors	416984	0.6%
Othman, 02 [97]			Convenience	Blood donors	2100	0.9%

MENA HIV ESP, Middle East and North Africa HIV/AIDS Epidemiology Synthesis Project database; PHIC, Palestinian Health Information Center; Prev, prevalence; Q, quarter; RDS, respondent driven sampling; SRS, simple random sampling; StRS, stratified random sampling; Trans, transfusion.

*The table reports only studies whose sample size is greater or equal to 50 participants. For space considerations, the table shows the overall HCV measure of each study rather than stratifications within population subgroups.

**The decimal places of the prevalence figures are as reported in the original report, but prevalence figures with more than one decimal places were rounded to one decimal place.

### Study selection

**Fig 1:**
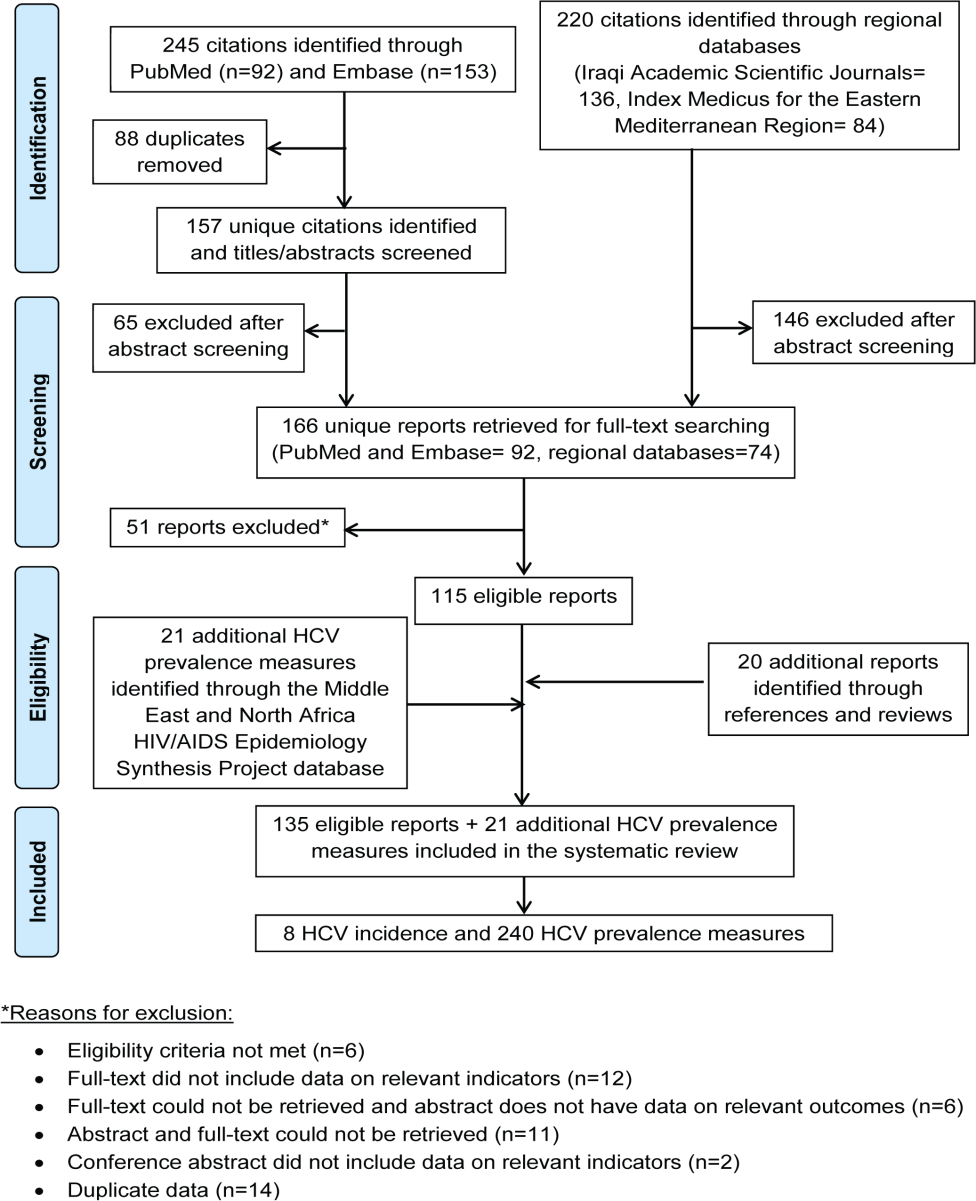
Flow chart of article selection for the systematic review of hepatitis C virus (HCV) incidence and prevalence in the Fertile Crescent countries, adapted from the PRISMA 2009 guidelines [16].

The search results were imported into a reference manager, Endnote, where duplicate publications were identified and excluded. The titles and abstracts of remaining records were screened for relevance, and full-texts of articles deemed relevant or potentially relevant were retrieved for further screening. The references of all articles and all reviews of literature were also checked to identify additional articles that could have been potentially missed. Any document reporting a measure of HCV incidence and/or prevalence based on primary data was considered for the review (Fig 1). Case reports, case series, editorials, letters to editors, commentaries, studies referring to hepatitis C as non-A non-B hepatitis, and records of HCV among military personnel who were stationed in this region for some time, but are from countries outside the region, were excluded from our review. A secondary independent screening for articles reporting HCV genotype information, irrespective of their inclusion of HCV incidence and/or prevalence measures, was also performed (S3 Fig).

In this work, a “report” refers to a document containing outcome measures of interest, while a “study” refers to all details pertaining to a specific outcome measure. Accordingly, one report could contribute multiple studies, and multiple reports of the same study, after being identified as duplicates, are considered as one study.

### Data extraction and data synthesis

Data from relevant reports were extracted by HC. To check for consistency, 10% of the reports were double extracted by another co-author (KC). The list of extracted information can be found in S1 Table. The extracted data were then synthesized by country and by populations’ risk of acquiring HCV (high, intermediate, and low risk). Our classification of risk was informed by HCV epidemiology literature [2, 22, 23] and a previously published classification [12]. In events where the level of exposure to HCV in certain populations was uncertain, and hence difficult to classify within the three pre-determined levels of risk, we followed a conservative approach that grouped such populations into a distinct risk category. We labeled this category as ‘special clinical populations’ since all of these populations were clinical populations. Details pertaining to the definitions and risk classification of identified populations can be found in S4 Fig.

The quality of HCV incidence and prevalence measures identified through our review was determined by assessing the risk of bias (ROB) for each study using the Cochrane approach [24], and by evaluating the precision of the reported measures. A description of the quality assessment criteria and studies’ ROB appraisal can be found in S5 Fig.

### Quantitative analysis

For each country, HCV antibody and RNA prevalence data in studies comprising at least 50 participants were stratified by population risk and summarized using reported ranges (Tables 2 and 3, and S2 and S3 Tables). Meta-analyses of HCV prevalence measures were conducted, for each country, for the various populations at risk whenever at least five HCV measures were available. Studies comprising a minimum of 25 participants were considered eligible for inclusion in the meta-analysis. HCV prevalence for the total sample was replaced with stratified measures whenever the sample size requirement was fulfilled at the level of each stratum. For each study, one final stratification was considered based on a pre-defined sequential order that prioritizes nationality followed by population, sex, year, region, and age.

The variance of the prevalence measures was stabilized using the Freeman-Tukey type arcsine square-root transformation [25]. Estimates for HCV prevalence were weighed using the inverse variance method and then pooled using a DerSimonian-Laird random effects model [26]. This model assumes a normal distribution of true effect sizes across studies, and therefore accounts for the sampling variation as well as the heterogeneity in effect size [27]. Heterogeneity measures were also calculated (Table 4) [27, 28].

**Table 4:** Pooled mean estimates for hepatitis C virus (HCV) prevalence stratified by populations’ risk of infection across countries of the Fertile Crescent.

	Studies	Samples	Prevalence	Effect size		Heterogeneity measures			
	Total N	Total N	Range (%)	Mean (%)	95% CI	Q (p-value)*	*τ* ^2^ **	I^2^ (confidence limits)^†^	Prediction interval (%)^‡^
**Iraq**									
General population	99	1859563	0.0–7.2	0.2	0.1–0.3	2355.9 (p<0.0001)	0.0014	95.8% (95.3–96.3%)	0.0–0.8
Populations at high risk	58	6707	0.0–67.3	19.5	14.9–24.5	1345.8 (p<0.0001)	0.1982	95.8% (95.1–96.4%)	0.0–62.8
Populations at intermediate risk	28	8398	0.0–35.1	1.5	0.7–2.5	205.2 (p<0.0001)	0.0227	86.8% (82.1–90.3%)	0.0–8.2
Special clinical populations	45	18845	0.0–76.2	5.0	3.4–6.9	1146.5 (p<0.0001)	0.0631	96.2% (95.5–96.7%)	0.0–22.1
**Jordan**									
General population	12	126152	0.0–2.0	0.3	0.1–0.5	170.7 (p<0.0001)	0.0016	93.6% (90.5–95.6%)	0.0–1.1
Populations at high risk	12	2888	21.0–59.5	37.0	29.3–45.0	137.8 (p<0.0001)	0.07	92.0% (87.9–94.7%)	11.1–67.7
Populations at intermediate risk	1	152	0.65						
Special clinical populations	1	143	40.5						
Mixed populations	1	426	4.5						
**Lebanon**									
General population	16	38059	0.0–3.4	0.2	0.1–0.3	38.2 (p<0.0008)	0.0008	60.8% (32.3–77.3%)	0.0–0.7
Populations at high risk	10	1309	0.0–52.8	14.5	5.6–26.5	223 (p<0.0001)	0.2011	96.0% (94.2–97.2%)	0.0–66.0
Populations at intermediate risk	7	1050	0.0–7.7	1.2	0.1–3.3	24.9 (p<0.0003)	0.0224	76.0% (49.3–88.6%)	0.0–10.7
Special clinical populations	4	312	0.0–19.6						
**Palestine**									
General population	53	337384	0.0–9.0	0.2	0.2–0.3	637.8 (p<0.0001)	0.0019	91.8% (90.1–93.3%)	0.0–0.9
Populations at high risk	4	584	6.8–45.2						
Populations at intermediate risk	0								
Special clinical populations	0								
Mixed populations	12	53363	0.3–4.9						
**Syria**									
General population	17	1114550	0.3–0.9	0.4	0.4–0.5	216.6 (p<0.0001)	0.0002	92.6% (89.7–94.7%)	0.3–0.7
Populations at high risk	8	1279	21.0–75.0	47.4	32.5–62.5	190.6 (p<0.0001)	0.1779	96.3% (94.5–97.6%)	3.8–93.8
Populations at intermediate risk	4	725	2.0–5.8						
Special clinical populations	2	218	1.0–48.0						
**All countries**									
General population	197	3475708	0.0–9.0	0.2	0.2–0.3	4497.5 (p<0.0001)	0.0013	95.6% (95.3–96.0%)	0.0–0.8
Populations at high risk	92	12767	0.0–75.0	23.6	19.8–27.5	2273.6 (p<0.0001)	0.1762	96.0% (95.5–96.4%)	0.3–64.5
Populations at intermediate risk	40	10325	0.0–35.2	1.5	0.9–2.3	243.5 (p<0.0001)	0.0208	84.0% (79.0–87.8%)	0.0–7.7
Special clinical populations	52	19518	0.0–76.2	5.4	3.7–7.4	1377.9 (p<0.0001)	0.0729	96.3% (95.7–96.8%)	0.0–24.4
Mixed populations	13	53789	0.3–4.9						

*Q: the Cochran’s Q statistic is a measure assessing the existence of heterogeneity in effect size.

**τ ^2^: the estimated between-study variance in the double arcsine transformed proportions of the true effect sizes. The back-transformed *τ* ^2^ was not calculated as the methodology to do so is not currently available.

^†^I^2^: a measure assessing the magnitude of between-study variation that is due to differences in effect size across studies rather than chance.

^‡^Prediction interval: estimates the 95% interval in which the true effect size in a new HCV study will lie.

For each country, we calculated the frequency of occurrence of each genotype relative to all identified genotypes (Table 5). Here, individuals testing positive for multiple genotypes contributed separately to the quantification of each of the identified genotypes. Shannon Diversity Index [29] was applied to the resulting distributions to assess the diversity of genotypes across countries, with a higher score indicating more diversity (Table 5). The country-specific distributions of HCV genotypes among HCV RNA positive individuals were also described (S6 Fig).

**Table 5:** Frequency, distribution and Shannon Diversity Index of identified hepatitis C virus (HCV) genotypes across countries of the Fertile Crescent.

Country	Iraq	Jordan	Lebanon	Palestine	Syria
	n (%)	n (%)	n (%)	n (%)	n (%)
**Genotype 1**	212 (33.2%)	30 (42.3%)	134 (39.9%)	38 (28.6%)	199 (29.5%)
**Genotype 2**	3 (0.5%)		53 (15.8%)	1 (0.8%)	5 (0.7%)
**Genotype 3**	24 (3.8%)		40 (11.9%)	2 (1.5%)	11 (1.6%)
**Genotype 4**	399 (62.5%)	41 (57.7%)	108 (32.1%)	92 (69.2%)	392 (58.2%)
**Genotype 5**			1 (0.3%)		67 (9.9%)
**Genotype 6**					
**Genotype 7**					
***Shannon Diversity Index (H)***	0.81	0.68	1.29	0.71	1.00
***Index relative to total possible diversity***	41.5%	35.0%	66.5%	36.6%	51.8%

Analyses were performed using SPSS 22.0 [30] and R 3.1.2. [31].

## Results

### Search results

Fig 1 describes the process of study selection based on PRISMA guidelines [16]. We identified a total of 465 citations (92 through PubMed, 153 through Embase, and 220 through regional databases). A total of 166 records were identified as relevant or potentially relevant after removing duplicates and screening the titles and abstracts of remaining records. Out of these, 115 records were eligible for inclusion in the systematic review during the full-text screening stage. Twenty additional records were identified through screening the bibliographies of studies and reviews. Twenty one HCV prevalence measures from country-level data were also identified through the MENA HIV/AIDS Epidemiology Synthesis Project database [18, 32–38]. To sum, a total of 135 eligible reports and 21 HCV prevalence measures from county-level data were included in the systematic review. These yielded 8 HCV incidence/sero-conversion and 240 HCV prevalence measures.

All 465 citations identified through our search underwent an independent secondary screening for HCV genotype studies (S3 Fig). A total of 163 records were identified as relevant or potentially relevant after removing duplicates and screening the titles and abstracts of remaining records. Out of these, 29 records were eligible for inclusion in this secondary systematic review during the full-text screening stage yielding 47 HCV genotype study measures.

### HCV incidence overview

Out of all countries being analyzed (Iraq, Jordan, Lebanon, Palestine, and Syria), data on HCV incidence was only available for Iraq and Jordan. There were no studies reporting strictly HCV incidence rate per person-year. All eight identified incidence measures were based on risk of sero-conversion over a specified time frame (Table 1). One study conducted across all dialysis units in Jordan reported a sero-conversion risk of 9.2% over one year [39]. The rest of the studies were conducted in Iraq. In one study among hemodialysis patients, a sero-conversion risk of 40.3% over one year was reported [40]. Lower HCV sero-conversion risks ranging from 0–3.5% were observed among pediatric cancer patients [41–43]. A study among newborns to HCV infected mothers reported 100% clearance of maternal antibodies by six months of age [44].

### HCV prevalence overview

We present here an overview of HCV prevalence studies identified in Iraq, Jordan, Lebanon, Palestine, and Syria. For the purpose of this analysis, we excluded HCV prevalence studies that were based on samples of less than 50 participants (37 studies). Accordingly, we report on a total of 203 HCV prevalence studies including 125 studies from Iraq, 15 from Jordan, 27 from Lebanon, 17 from Palestine, and 19 from Syria.

#### Iraq

Among populations at high risk (Table 2), high HCV prevalence levels ranging from 4% to 67.3% were reported among thalassemic patients, with the highest prevalence being observed among the 2 to 10 year-olds [45]. High HCV prevalence levels were also found among hemodialysis patients (range: 0–42.6%) [46–55] and hemophilia patients (range: 6.7–40%) [56–58].

For the general population (Table 3), HCV prevalence varied between 0% and 5.1%, and ranged between 0% and 2.8% among blood donors, 0% and 5.1% among pregnant women, and was 0.4% in a national household-based survey [59] and overall less than 1% among other general population groups.

Among populations at intermediate risk (S2 Table), HCV prevalence ranged from 0% to 3.3% among health care workers [46, 50, 60–64], and was higher among dental providers (9.1%) [65], hospitalized patients (14%) [66], and type I diabetes mellitus patients (35.1%) [67]. HCV prevalence also varied widely among special clinical populations ranging from 0% among patients with malignancies or liver-related problems to 71.9% among acute viral hepatitis patients (S2 Table).

#### Jordan

Among populations at high risk (Table 2), high HCV prevalence levels ranging from 21% to 49.8% were observed among hemodialysis patients. A prevalence of 32.8% was also observed among thalassemic patients [68].

For the general population (Table 3), low HCV prevalence levels of less than 1% were observed among blood donors and people attending health centers (range: 0.1–0.9%). Among populations at intermediate risk and special clinical populations (S2 Table), low HCV prevalence of 0.6% was reported among health care workers [69] and high prevalence of 40.5% was reported among patients with hemolytic anemia [70].

#### Lebanon

Among populations at high risk (Table 2), high HCV prevalence of 52.8% was reported among PWID [71]. Overall high HCV prevalence was also reported among other populations at high risk (Table 2) such as hemodialysis patients (16% [72] and 27% [73]), thalassemia patients (0% [74] and 14% [75]), and multi-transfused cancer patients (4.6% [76]).

For the general population (Table 3), HCV prevalence was measured at less than 0.6% among Lebanese nationals [73, 76–84] and at about 3% among non-Lebanese nationals residing in Lebanon [83, 84].

Rather low HCV prevalence was observed among populations at intermediate risk ranging from 0% among female sex workers and men who have sex with men to 7.7% among HIV patients infected apparently via the sexual route (S2 Table). Among special clinical populations (S2 Table), HCV prevalence was 19.6% [85] among hepatocellular carcinoma patients and 0% [82, 86] among patients with other malignancies.

#### Palestine

Among populations at high risk (Table 2), HCV prevalence was measured at 27.4% among hemodialysis patients in the West Bank [87], and at 17.9% among hemodialysis patients in the Gaza strip [88]. A prevalence of 45.2% was reported among PWID in East Jerusalem [89].

For the general population (Table 3), HCV prevalence among blood donors from different parts of Palestine ranged between 0.2% and 0.3% [18, 32–38]. In older studies, all of which were in the Gaza strip, HCV prevalence was reported at 2.2% and 3.9% among blood donors [90, 91] and at 9% among outpatient hospital attendees [90].

#### Syria

Among populations at high risk (Table 2), high HCV prevalence ranging from 48.9% to 75% was reported among hemodialysis patients [92–94]. Meanwhile, HCV prevalence among drug users (injecting and non-injecting) and among hemophilia patients was measured at 21% [95] and 20.5% [96], respectively.

For the general population (Table 3), HCV prevalence ranged between 0.3% and 0.9% among blood donors. Among populations at intermediate risk (S2 Table), HCV prevalence ranged between 2% among female sex workers [97] and up to 3.8% among health care workers [98]. The only study among special clinical populations (S2 Table) was among acute viral hepatitis patients and reported a prevalence of 1.0% [99].

#### Overall HCV prevalence ranges and medians

For all countries, HCV prevalence ranged from 0–75% among populations at high risk with a median of 25.0%, from 0–9% among populations at low risk with a median of 0.4%, from 0–35.1% among populations at intermediate risk with a median of 1.2%, and from 0–71.9% among special clinical populations with a median of 3.4%.

### Pooled mean HCV prevalence estimates

Our pooled country-specific estimates for the national population-level HCV prevalence, based on pooling the general population measures, were: 0.2% (95% CI: 0.1–0.3%) in Iraq, 0.3% (95% CI: 0.1–0.5%) in Jordan, 0.2% (95% CI: 0.1–0.3%) in Lebanon, 0.2% (95% CI: 0.2–0.3%) in Palestine, 0.4% (95% CI: 0.4–0.5%) in Syria, and 0.2% (95% CI: 0.2–0.3%) in FC countries as a whole (Table 4). The prediction interval for each of these pooled means, that is the estimated 95% interval range for the true effect size (HCV prevalence) across studies, also rarely exceeded 1% (Table 4). The forest plots of the country-specific meta-analyses can be found in S7–S9 Figs.

High pooled HCV prevalence levels were estimated for populations at high risk: 19.5% (95% CI: 14.9–24.5%) in Iraq, 37.0% (95% CI: 29.3–45.0%) in Jordan, 14.5% (95% CI: 5.6–26.5%) in Lebanon, 47.4% (95% CI: 32.5–62.5%) in Syria, and 23.6% (95% CI: 19.8–27.5%) in FC countries as a whole (Table 4). The prediction intervals for these measures were also wide across countries (Table 4).

For populations at intermediate risk, the pooled HCV prevalence was 1.5% (95% CI: 0.7–2.5%) in Iraq, 1.2% (95% CI: 0.1–3.3%) in Lebanon, and 1.5% (95% CI: 0.9–2.3%) in FC countries as a whole (Table 4). For special clinical populations, the pooled HCV prevalence was 5.0% (95% CI: 3.4–6.9%) in Iraq and 5.4% (95% CI: 3.7–7.4%) in FC countries as a whole (Table 4). The prediction intervals for these measures were also wide across countries (Table 4).

There was significant evidence for heterogeneity in effect size in all meta-analyses, the p-value was always <0.001 (Table 4). Most of the variation in all meta-analyses was attributed to variation in effect size across studies rather than chance (I^2^ >60%; Table 4).

### HCV RNA prevalence

Our search identified a total of 34 HCV RNA measures (S3 Table). Generally high HCV RNA prevalence was found among HCV antibody positive individuals at high risk across all countries ranging from 26.1% among hemodialysis patients to 88% among thalassemia patients. HCV RNA prevalence was also substantial among HCV antibody negative individuals at high risk ranging from 3.5% among hemodialysis patients to 16.7% among thalassemic children.

For the general population (S3 Table), the prevalence of HCV RNA among HCV antibody positive individuals in Iraq and Palestine hovered around 65% in different samples of blood donors [90, 100, 101], pregnant women [102], and outpatients [90], but was at 89.7% among blood donors in Jordan [103].

### HCV genotypes

Information on HCV genotypes was available in 47 studies including a total of 1749 HCV RNA positive individuals (Table 5 and S6 Fig).

In Iraq, the majority of participants were infected by a single strain, only 11.3% had multiple genotypes isolated from their plasma (S6A Fig) [45, 48, 101, 102, 104–110]. The distribution of HCV genotypes showed a high prevalence for genotype 4 (62.5%) and genotype 1 (33.2%) followed by genotype 3 (3.8%) and genotype 2 (0.5%) (Table 5). Subtypes 1a and 1b were isolated, respectively, from 33.2% and 66.8% of infected persons with genotype 1 (where subtype information was available). About two-third of the infections among populations at high risk and slightly more than half of those among general populations were attributed to genotype 4.

All HCV RNA positive individuals in Jordan were infected by a single strain (S6B Fig), with 57.7% carrying genotype 4 and 42.3% carrying genotype 1 (Table 5) [68, 103, 111, 112]. Subtypes 1a and 1b were isolated, respectively, from 54.5% and 45.5% of infected persons with genotype 1. The majority of genotype 1 samples were among hemodialysis and thalassemia patients, while samples with genotype 4 were equally distributed between populations at high risk and the general population.

In Lebanon, over 90% of participants were infected by a single strain (S6C Fig) [71, 72, 75, 76, 113–117]. The distribution of genotypes indicated genotype 1 as the most common (39.9%), followed by genotype 4 (32.1%), genotype 2 (15.8%), genotype 3 (11.9%) and genotype 5 (0.3%) (Table 5). Studies reporting subtype information for genotype 1 showed an equal distribution of subtypes 1a and 1b. About half of genotype 3 infections (47.5%) were collected among PWID; and an additional third were collected among hemodialysis and thalassemia patients. Genotype 3 was also identified in a prison setting [113]. Genotype 5 was reported in only one study among a sample of patients with symptomatic liver disease [116].

All HCV genotype studies in Palestine were conducted in the Gaza strip [90, 118]. Almost all infections were single strained (S6D Fig). Genotype 4 was the most common (69.2%), followed by genotype 1 (28.6%), genotype 3 (1.5%) and genotype 2 (0.8%) (Table 5). Subtypes 1a and 1b constituted, respectively, 69.0% and 31.0% of genotype 1 cases.

The distribution of HCV genotypes in Syria (S6E Fig) revealed genotype 4 as the most common (58.2%) followed by genotype 1 (29.5%), genotype 5 (9.9%), genotype 3 (1.6%) and genotype 2 (0.7%) (Table 5) [92, 119, 120]. Subtypes 1a and 1b were isolated, respectively, from 44.4% and 55.6% of infected persons with genotype 1.

The largest possible value for the Shannon Diversity Index, assuming equal distribution of the seven known HCV genotypes [121], is 1.95 [29]. Lebanon scored highest on this index (H = 1.29 out of 1.95 or 66.5%) indicating high diversity in the types and frequency of isolated genotypes (Table 5). The next most diverse genotype distributions were found in Syria (H = 1.00 out of 1.95 or 51.8%), and Iraq (H = 0.81 out of 1.95 or 41.5%), while the least diversity was observed in Palestine, more specifically in the Gaza strip (H = 0.71 out of 1.95 or 36.6%), and Jordan (H = 0.68 out of 1.95 or 35.0%).

### Risk factors for HCV infection

Few studies (n = 5) investigated possible risk factors for HCV infection using multivariable logistic regression analyses. Common reported risk factors among hemodialysis patients included duration of dialysis and number of blood units transfused [54, 94]. Among individuals presenting to laboratory units for diverse testing in Lebanon, hemodialysis was the main risk factor for HCV infection [84]. Meanwhile, maternal age and number of previous miscarriages were identified as the main risk factors for HCV infection among pregnant women in Iraq [102]. Among patients suspected of having acute viral hepatitis, blood transfusion, tattooing, and cupping were identified as risk factors for HCV infection in a study in Iraq [122].

### Quality assessment of HCV incidence and prevalence measures

The results of the quality assessment conducted for the HCV incidence and prevalence measures are summarized in S4 Table. The study-specific quality assessment for each of these measures is found in S5–S8 Tables.

The majority of HCV incidence studies involved samples of less than 100 patients, and thus were classified as having low precision. All studies were based on convenience samples selected from clinical facilities and were characterized by a high response rate. The type and generation of the biological assays used to ascertain infection were specified in almost all studies, with more than half relying on the more recent, sensitive and specific 3^rd^ and 4^th^ generation ELISA tests.

The majority of HCV prevalence measures had high precision (81.3%). The ROB assessment was possible for 184 HCV prevalence measures, while 19 measures were judged to be of unknown quality as limited description of the samples was available and were excluded from further analyses. However, all studies with unknown ROB were based on testing among blood donors reported by ministries of health and blood banks. For the vast majority of measures (98.4%), HCV ascertainment was based on biological assays. Information about the generation of the used assay was missing for more than half of the studies. Among studies with information on the assay’s generation, 85.2% reported the use of the more sensitive and specific 3^rd^ and 4^th^ generation assays. Most of the studies were drawn using convenience sampling. Half of the studies had low ROB in the response rate domain, while 3.8% had high ROB. Response rate information was missing for 46.2% of the measures.

Overall, HCV incidence and prevalence studies were of reasonable quality, but incidence studies were of lower precision. Nearly all studies had low ROB in at least one quality domain (99.0%), and 47.9% of all studies had low ROB in at least two of the three quality domains. Less than 3.6% of all studies had high ROB in two or three quality domains.

## Discussion

We presented to our knowledge the first systematic review and synthesis of HCV incidence and prevalence across countries of the FC subregion of MENA. We also provided estimates for HCV prevalence at the national level and for various at risk populations. Our results suggest that HCV prevalence is at less than 1% among the general population across these countries (Table 4). Meanwhile, high prevalence levels were noted among hemodialysis, thalassemia and multi-transfused patients (Table 2). Genotypes 4 and 1 seem to be the most common genotypes circulating in these countries (Table 5 and S6 Fig).

Our estimates for the national HCV prevalence of less than 1% suggest that HCV levels in these countries are comparable to those in developed and other developing countries, but are towards the lower end of the global range [1, 2, 123]. These estimates also suggest that this subregion appears to have the lowest HCV prevalence of all MENA subregions [7, 11–15]—substantially lower than in other MENA countries namely Egypt [6, 7] and Pakistan [8, 9].

A large fraction of HCV prevalence measures in the general population were among blood donors, and HCV prevalence in this normally healthy population may underestimate HCV prevalence in the population at large. In the USA, for example, HCV prevalence among blood donors [124] is substantially lower than that estimated in nationally-representative population-based surveys [125]. We conducted sensitivity analyses (not shown) to examine the impact of excluding blood donor data on our pooled estimates for HCV prevalence. For Iraq, HCV prevalence increased substantially from 0.2% to 0.8% with the exclusion of blood donors. Meanwhile, there were minor changes to the prevalence in Jordan (0.3% versus 0.2%) and Lebanon (0.2% versus 0.3%). Sensitivity analyses were not possible for Palestine and Syria due to the small number of studies among other general population groups (1 study in Palestine and 0 studies in Syria).

Overall, these results support our conclusion that HCV prevalence is likely to be below 1% among the population at large in each of the FC countries. It bears notice that our estimate for HCV prevalence in Iraq is substantially lower than that estimated recently using a different methodology in a global study (0.2% in our study versus 3.2% in *Gower et al*. study [22]). The difference in estimates arises from *Gower et al*. [22] basing their estimate on a single study [102] while our pooled prevalence was based on 99 studies, a large fraction of which were identified through our expanded search of data in non-indexed journals.

Despite the lower prevalence among the population at large, our findings indicate on-going transmission in at least some medical facilities. Exposures to hemodialysis [54, 84, 94] or blood transfusion [54, 122] were cited as risk factors for acquiring HCV. High HCV prevalence was generally observed among hemodialysis and thalassemia patients across countries (Table 2). High sero-conversion risks of up to 40% were found among hemodialysis patients in prospective studies (Table 1). Measurable HCV RNA prevalence among HCV antibody negative clinical populations were documented suggesting recent nosocomial HCV exposures (S3 Table). Higher HCV levels (compared to the general population) among health care workers, hospitalized populations, different clinical populations, diabetics, and dental providers were identified suggesting health care related HCV exposures (S2 Table). While some of the prevalent HCV exposures may date to times before the enforcement of more stringent blood screening and infection control protocols in these countries, the totality of the evidence supports the conclusion of ongoing transmission in some clinical settings and through medical procedures. These results emphasize the importance of reinforcing blood screening and infection control in health care facilities.

Other HCV risk factors identified in this review included exposure to infected needles and sharp objects during tattooing and cupping procedures [122]. The latter, also known as *hijama*, is a common practice by community healers in MENA [18, 126]. There is also evidence elsewhere in MENA of a wider scope of community related HCV exposures as well as traditions of some professions, such as barbers, administering health care related procedures such as injections [7, 18, 127]. These findings highlight the relevance of controlling these exposures and insuring the safety of injections. This could be done, for example, by wide-scale replacement of current re-usable syringes with safety-engineered “smart” syringes [128, 129].

The ongoing political and military conflicts in this subregion (Iraq, Palestine, and Syria) introduce more challenges and serious barriers to the implementation of stringent blood screening and infection control in medical facilities and in communities. Shortages of medical supplies and trained human capital in these countries are often exacerbated by intermittent electricity and water supplies making the provision of even basic medical care difficult [42, 130–132]. Implications of these emergencies on current HCV transmission are unknown.

Injecting drug use appears to be the dominant driver of HCV incidence in many developed countries [133–135]. The estimated population proportion of injecting drug use among the adult population in FC ranges between 0.07% in Syria and 0.35% in Palestine [136]. The population proportion of injecting drug use in MENA as a whole is estimated at 0.24%; in the intermediate range compared to global levels [136]. Our search identified only few studies assessing the status of HCV infection among PWID and other drug users [18, 71, 89]. These showed HCV prevalence varying between 21% in Syria and 52.8% in Lebanon (Table 2). Relatively low HCV prevalence has been observed among prisoners, a fraction of whom are often drug users, in Iraq (0.6%) [137] and in Lebanon (3.4%) [113]. HCV prevalence among PWID in FC seems thus somewhat lower than that observed in other MENA countries and globally [23, 136]. With these overall limited data on HCV among PWID, it is difficult to judge the relative importance of injecting drug use as a driver of HCV incidence and prevalence in FC.

Migration dynamics within MENA may have played a role in HCV epidemiology in this subregion. This is specially so in relation to the epidemic in Egypt, the country with the largest HCV prevalence worldwide [6, 7]. The epidemic in Egypt is almost exclusively dominated by genotype 4 (>90%) [138], which is also a highly prevalent genotype in FC (Table 5). Genotype 4 in MENA appears to have a larger frequency in countries geographically close to Egypt and/or in countries that host or have hosted large migrant labor populations from Egypt [11, 12]. Iraq in particular, but also Jordan and Lebanon, have hosted or are currently hosting millions of Egyptian migrant labor [139–142]. A large fraction of the labor market in Iraq at some point consisted of Egyptian migrant workers who formed the backbone of the major infrastructure and developmental projects and agricultural sector in this country—such as during the era of the Iraq-Iran war [139–141]. Movement of Egyptian labor, who often came from rural areas most affected by HCV [7], may have contributed to higher circulation of genotype 4 in host countries, but has had a limited impact on HCV prevalence in these countries. FC countries appear to have some of the lowest HCV prevalence levels globally.

The Gaza strip, which borders Egypt, seems to be the part of FC most influenced by HCV dynamics in Egypt. The Gaza strip was under Egyptian rule prior to the 1967 Israeli occupation and shares strong historical, cultural, and economic ties with Egypt. With the protracted Israeli siege of Gaza, Egypt has become the main gateway for Gaza residents to the world and the country that provides essential services to the people of Gaza. Gazans often seek medical treatment in Egypt for conditions that cannot be treated in this largely impoverished district. It is probably not surprising therefore that genotype 4 appears to be the dominant genotype in Gaza, and specially so among those with a history of travel, or of receiving medical care, including blood transfusions or surgical operations, in Egypt [118].

A substantial fraction of genotype 3 infections in FC were observed among PWID, affirming a global link between this genotype and injecting drug use [121, 138, 143]. Apart from Lebanon, very limited number of genotype 3 infections were identified (Table 5). One reason could be that PWID contribution to HCV incidence is not large in these countries, and/or that genotypes 4 and 1 are the dominant genotypes circulating among PWID. An alternative explanation, and possibly a more plausible one, is that existing studies of genotypes have not yet reached this largely hidden population whose contribution to HCV incidence and prevalence remains poorly understood.

Lebanon and Syria showed higher genotype diversity with genotype 5 only detected in these countries, and genotype 2 being more prevalent in Lebanon than in the rest of FC (Table 5). These findings are consistent with the large scale emigration rates and diaspora populations of these two countries globally—specifically so for Lebanon. Such population movements may provide avenues for genotypes that are prevalent in other parts of the world, say in West Africa and Latin America for genotype 2 [121, 141] and South Africa for genotype 5 [121, 138], to be introduced into circulation in some of these countries.

Our systematic review and meta-analyses are limited by the variability in quantity and quality of studies across countries. There were few studies that assessed HCV incidence and some countries did not have sufficient number of prevalence measures in some of the at risk populations to warrant conduct of meta-analyses. With the convenience sampling in most studies, and the country-specific variability in frequency, specific population and geographical location of available data, the estimates may not be representative of the true HCV population prevalence. Data was also limited for some of the specific populations that play an important role in HCV epidemiology, most notably PWID. HCV RNA measures were limited in quantity and were often based on relatively small samples.

The number of genotyped infections was not large in all countries and available genotype data may not have captured the diversity and true frequency of genotypes across the different at risk populations in each country. For example, there were some differences, though minor, between the genotype frequencies identified here compared to those of two recent studies of global genotype distribution [22, 121]. The main source of differences appears to be the inclusion of more studies on HCV genotypes in our review, largely as a consequence of including regional and national databases in our search of non-indexed published literature (S3 and S6 Figs).

Our meta-analyses highlighted heterogeneity in effect size among included studies. This is not unexpected considering the differences in prevalence measures in terms of the specific population studied, sampling methodology and participant recruitment, age-group representation in the sample, year of study, location and assay used [12]. However, due to the relatively small number of outcome measures for each country, we were unable to conduct a meaningful multivariable meta-regression analysis to identify sources of heterogeneity in effect size.

## Conclusions

HCV prevalence in the population at large in FC appears to be below 1%, and is tending towards the lower end of the global range. HCV prevalence among high risk populations, such as hemodialysis and thalassemia patients, is considerable and the evidence indicates ongoing HCV transmission in at least some medical facilities. Genotypes 4 and 1 appear to be the most prevalent, and genotype distributions suggest a role for recent migrations in the circulation of different genotypes across national borders. With the limitations in available data, infection levels among PWID and the contribution of this population to HCV incidence and prevalence remain uncertain. The implications of the ongoing military conflicts and ensuing emergencies in FC on current HCV transmission are unknown.

Further research is needed for a better understanding of the epidemiology of this infection in this MENA subregion. Elucidation of the role of key populations in HCV transmission, such as PWID, is needed. Conduct of nationally-representative population-based surveys to estimate HCV prevalence, delineate spatial variability in prevalence, identify modes of exposure, and assess HCV knowledge and attitudes, as has been done recently in Egypt [6, 144–148], will enhance our knowledge of the epidemiology of this infection in this part of the world.

Our findings inform planning of health service provision, articulation of HCV policy guidelines, and implementation of effective HCV programming to reduce HCV transmission and decrease the burden of its associated diseases. Since HCV exposures in FC are linked to specific settings, HCV prevention efforts should be targeted and focus on infection control in clinical settings and harm reduction among PWID. Adoption of the new World Health Organization guidelines for the use of safety-engineered syringes [128, 129] can also minimize exposure to HCV and other blood-borne pathogens.

## Supporting Information

S1 Fig Preferred Reporting Items for Systematic Reviews and Meta-analyses (PRISMA) checklist. (TIF)

S2 Fig Data sources and search criteria for systematically reviewing hepatitis C virus (HCV) incidence and prevalence in countries of the Fertile Crescent. (TIF)

S3 Fig Flow chart of article selection for the systematic review of hepatitis C virus (HCV) genotypes in the Fertile Crescent countries, adapted from the PRISMA 2009 guidelines. (TIF)

S4 Fig Definitions and risk classification of populations identified through the systematic review. (TIF)

S5 Fig Description of quality assessment criteria and studies’ risk of bias (ROB) appraisal. (TIF)

S6 Fig Distribution of hepatitis C virus (HCV) genotypes and subtypes among HCV RNA positive individuals across countries of the Fertile Crescent. Data on individuals infected with multiple HCV genotypes was only available for Iraq, Lebanon and Palestine.
(TIF)

S7 Fig Forest plot presenting the results of the meta-analysis of hepatitis C virus (HCV) prevalence measures among the general population in Iraq. (TIF)

S8 Fig Forest plots presenting the results of the meta-analyses of hepatitis C virus (HCV) prevalence measures among the general population in: A) Jordan, B) Lebanon, and C) Syria. (TIF)

S9 Fig Forest plot presenting the results of the meta-analysis of hepatitis C virus (HCV) prevalence measures among the general population in Palestine. (TIF)

S1 Table Lists of variables extracted from relevant reports with hepatitis C virus (HCV) incidence and/or prevalence information or with HCV genotype information. (DOCX)

S2 Table Studies reporting hepatitis C virus (HCV) prevalence among populations at intermediate risk, special clinical populations, and mixed populations in countries of the Fertile Crescent. (DOCX)

S3 Table Studies reporting hepatitis C virus (HCV) RNA prevalence in countries of the Fertile Crescent. (DOCX)

S4 Table Summary of precision and risk of bias assessment for hepatitis C virus (HCV) incidence and prevalence measures extracted from eligible reports. (DOCX)

S5 Table Precision and risk of bias (ROB) assessment for individual hepatitis C virus (HCV) incidence measures in countries of the Fertile Crescent. (DOCX)

S6 Table Precision and risk of bias (ROB) assessment for individual hepatitis C virus (HCV) prevalence measures among populations at high risk in countries of the Fertile Crescent. (DOCX)

S7 Table Precision and risk of bias (ROB) assessment for individual hepatitis C virus (HCV) prevalence measures among the general population in countries of the Fertile Crescent. (DOCX)

S8 Table Precision and risk of bias (ROB) assessment of individual hepatitis C virus (HCV) prevalence measures among populations at intermediate risk and among special clinical populations in countries of the Fertile Crescent. (DOCX)
