# Supplementary figures and images for: The Epidemiology of Hepatitis C Virus in the Fertile Crescent: Systematic Review and Meta-Analysis
Source: PLoS One. 2015 Aug 21;10(8):e0135281. doi: 10.1371/journal.pone.0135281 (PMC4546629; doi:10.1371/journal.pone.0135281)

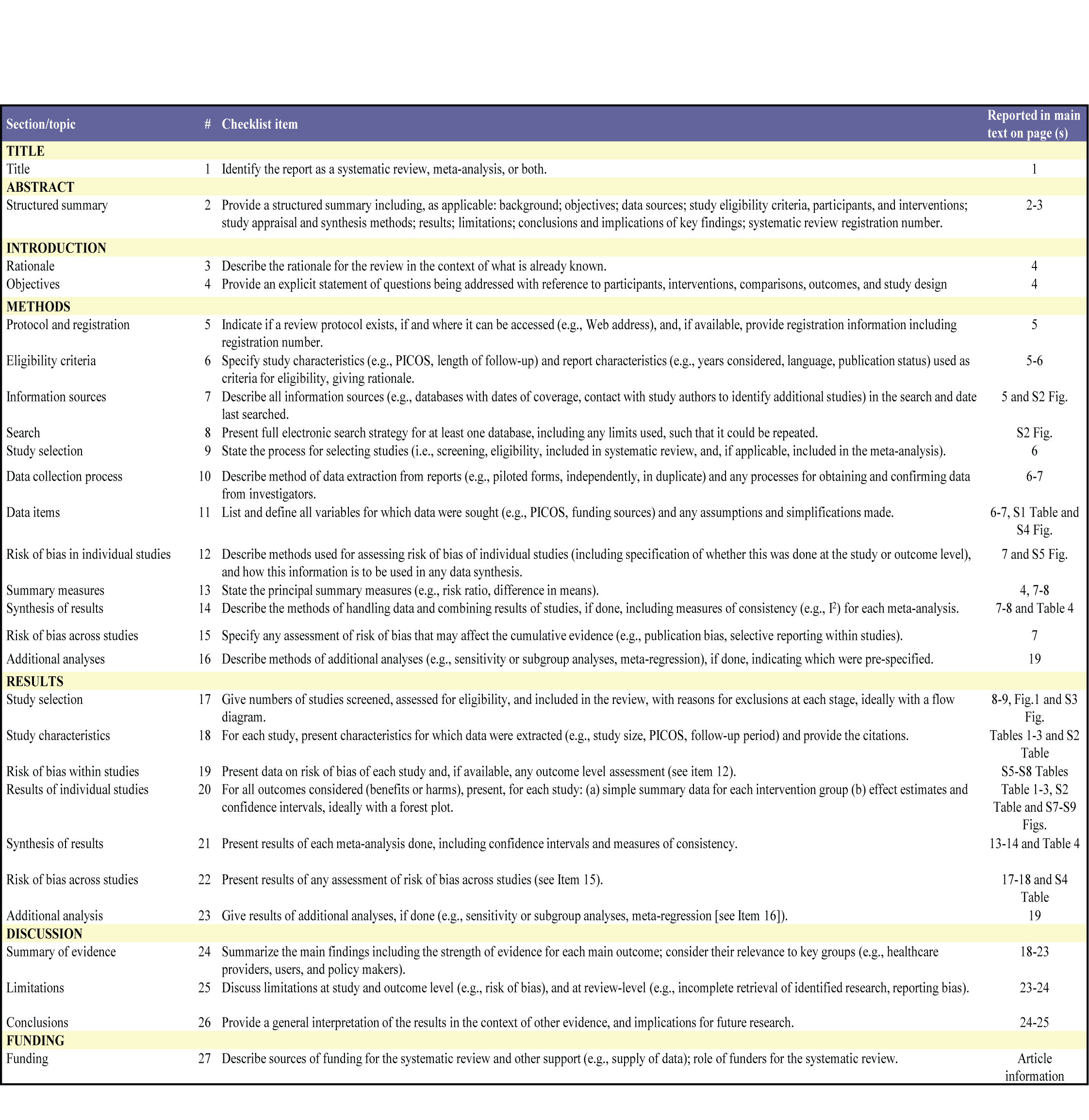

Supplement: S1 Fig — (TIF) [file pone.0135281.s001.tif]

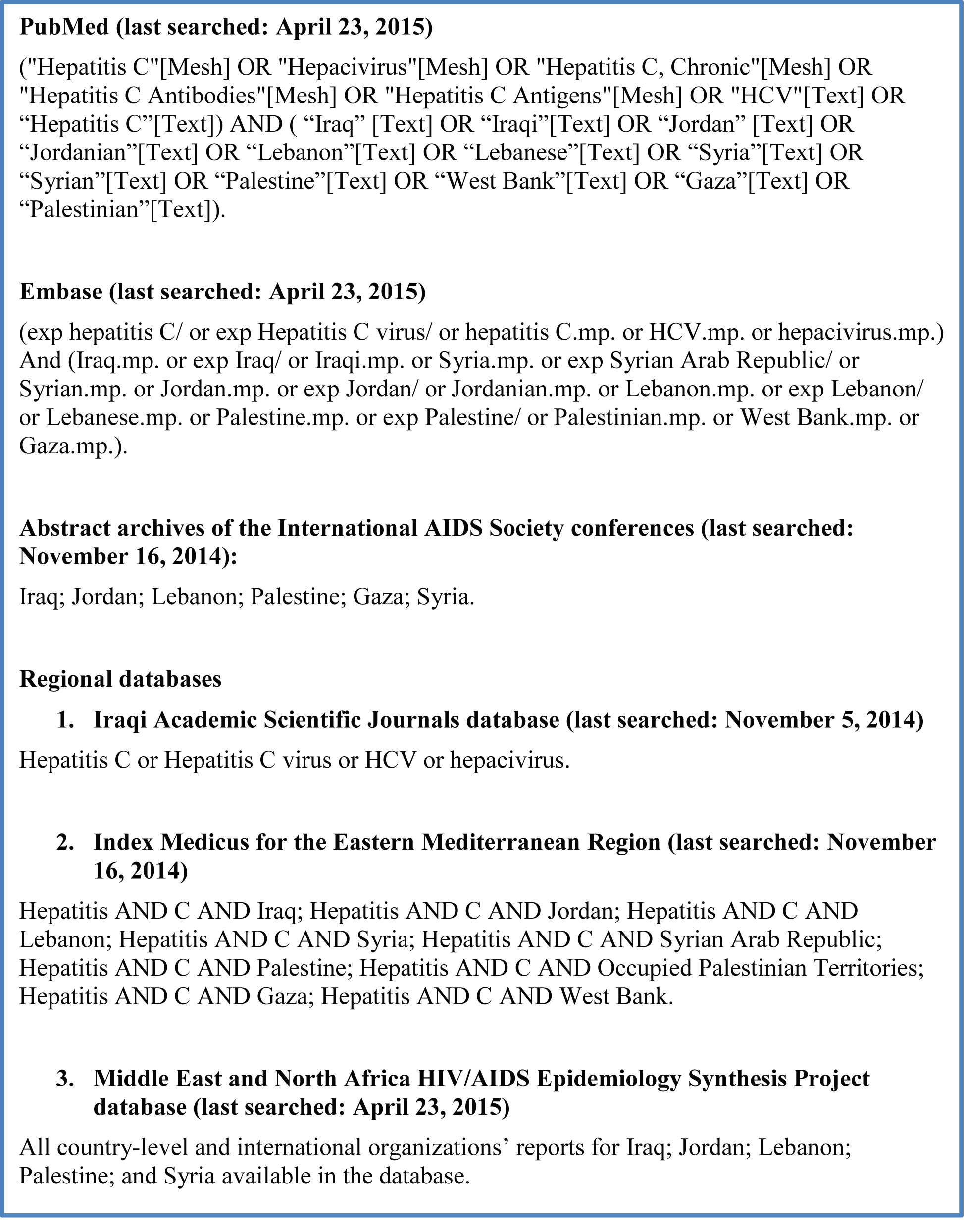

Supplement: S2 Fig — (TIF) [file pone.0135281.s002.tif]

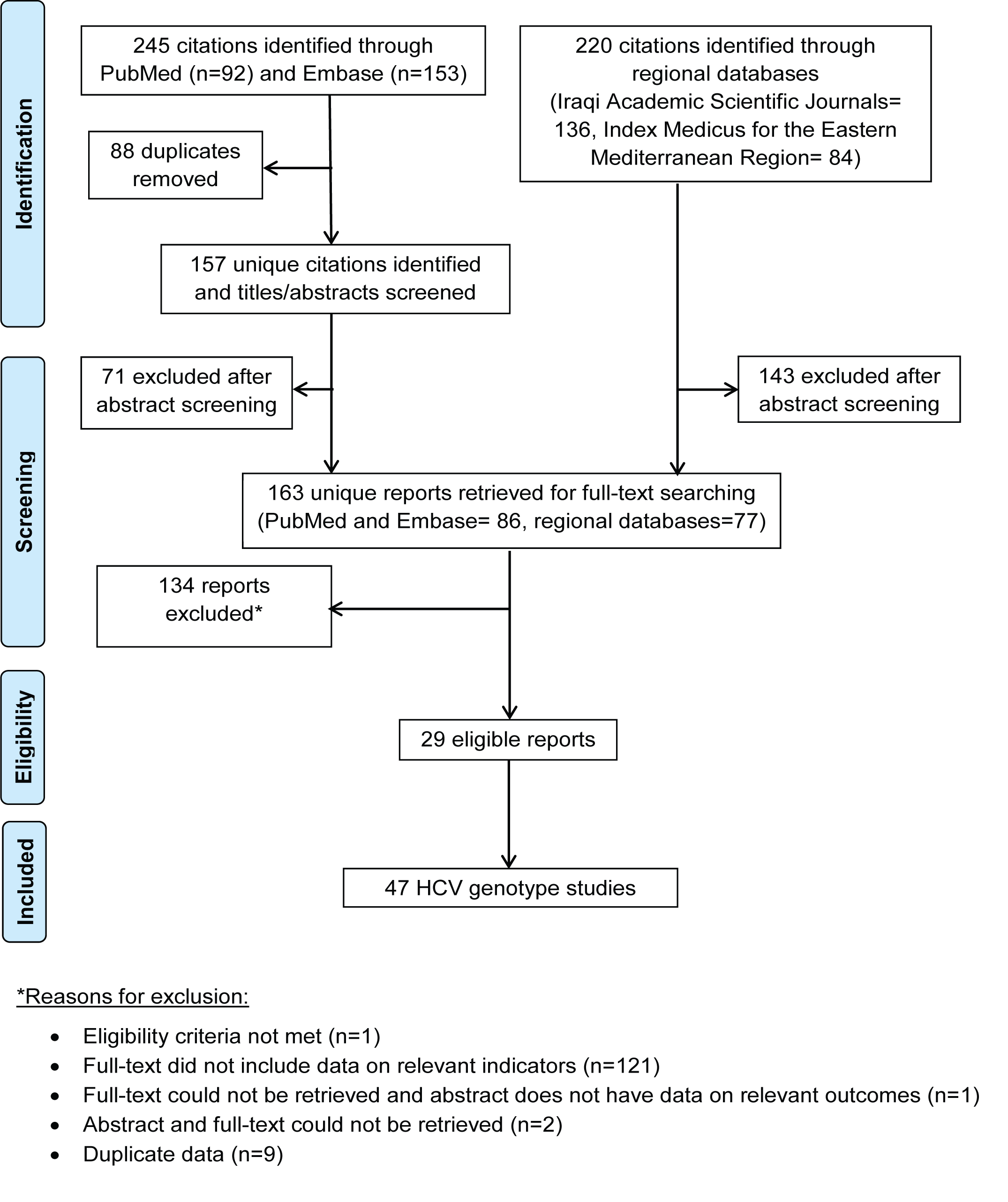

Supplement: S3 Fig — (TIF) [file pone.0135281.s003.tif]

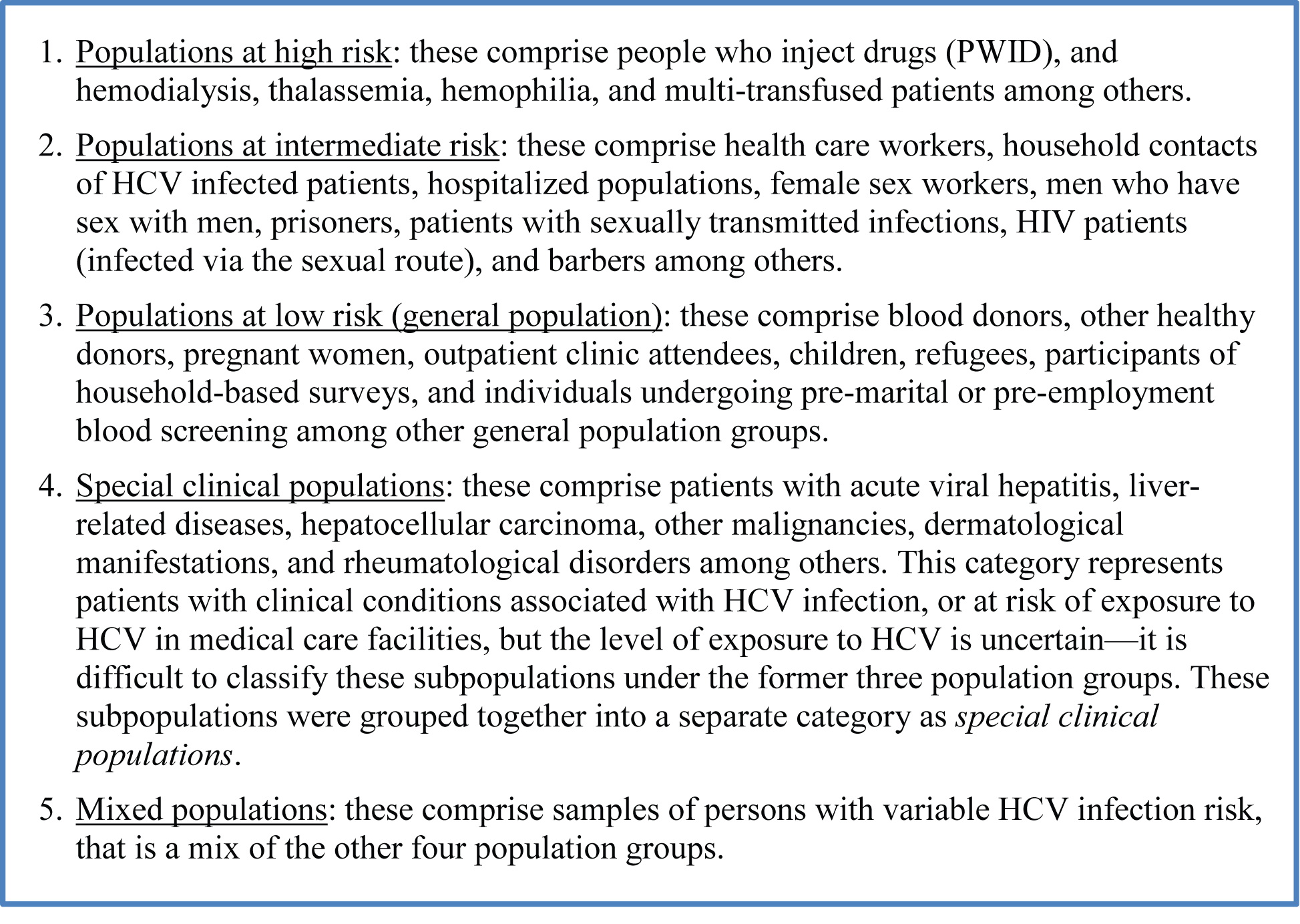

Supplement: S4 Fig — (TIF) [file pone.0135281.s004.tif]

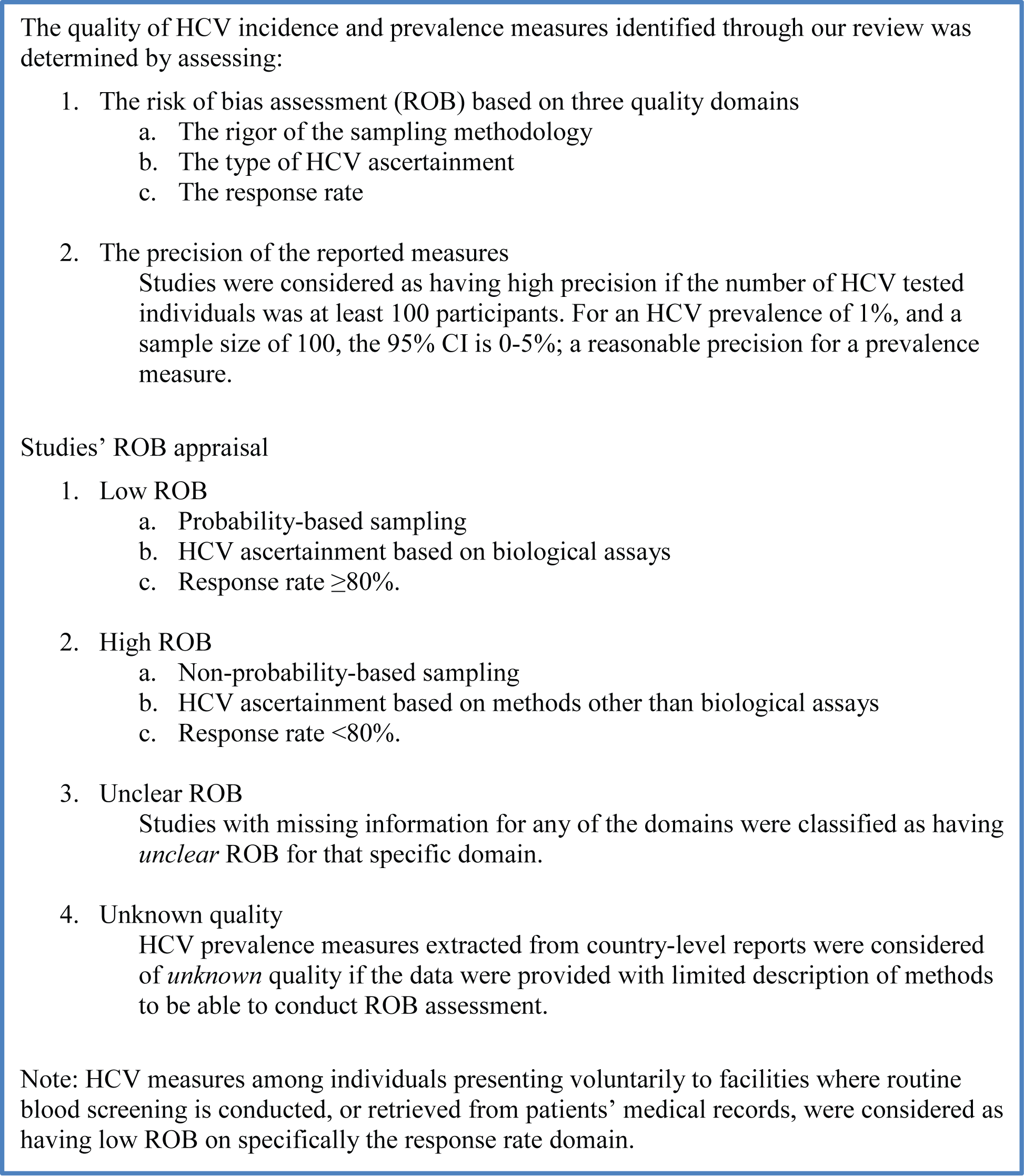

Supplement: S5 Fig — (TIF) [file pone.0135281.s005.tif]

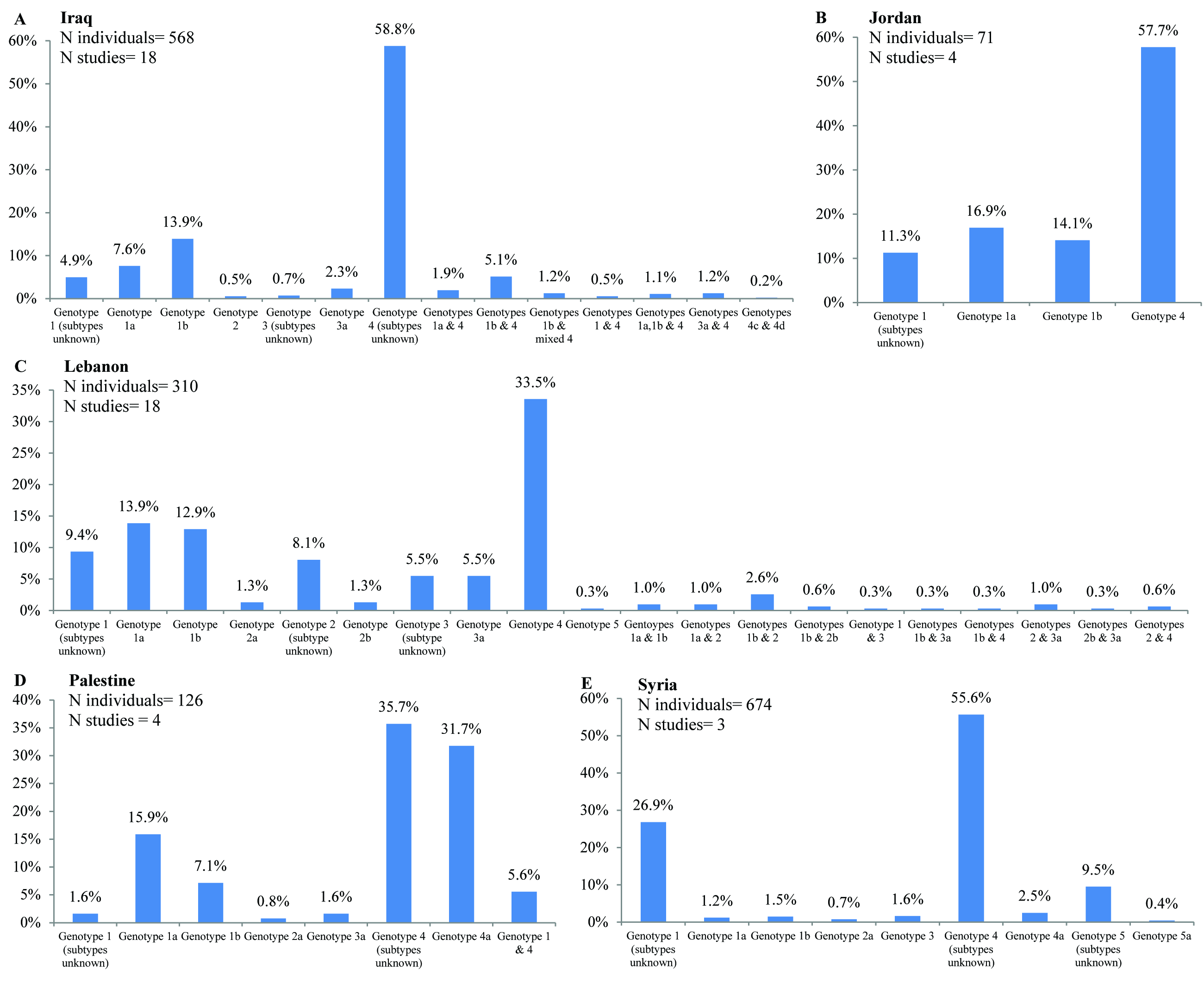

Supplement: S6 Fig — Data on individuals infected with multiple HCV genotypes was only available for Iraq, Lebanon and Palestine. (TIF) [file pone.0135281.s006.tif]

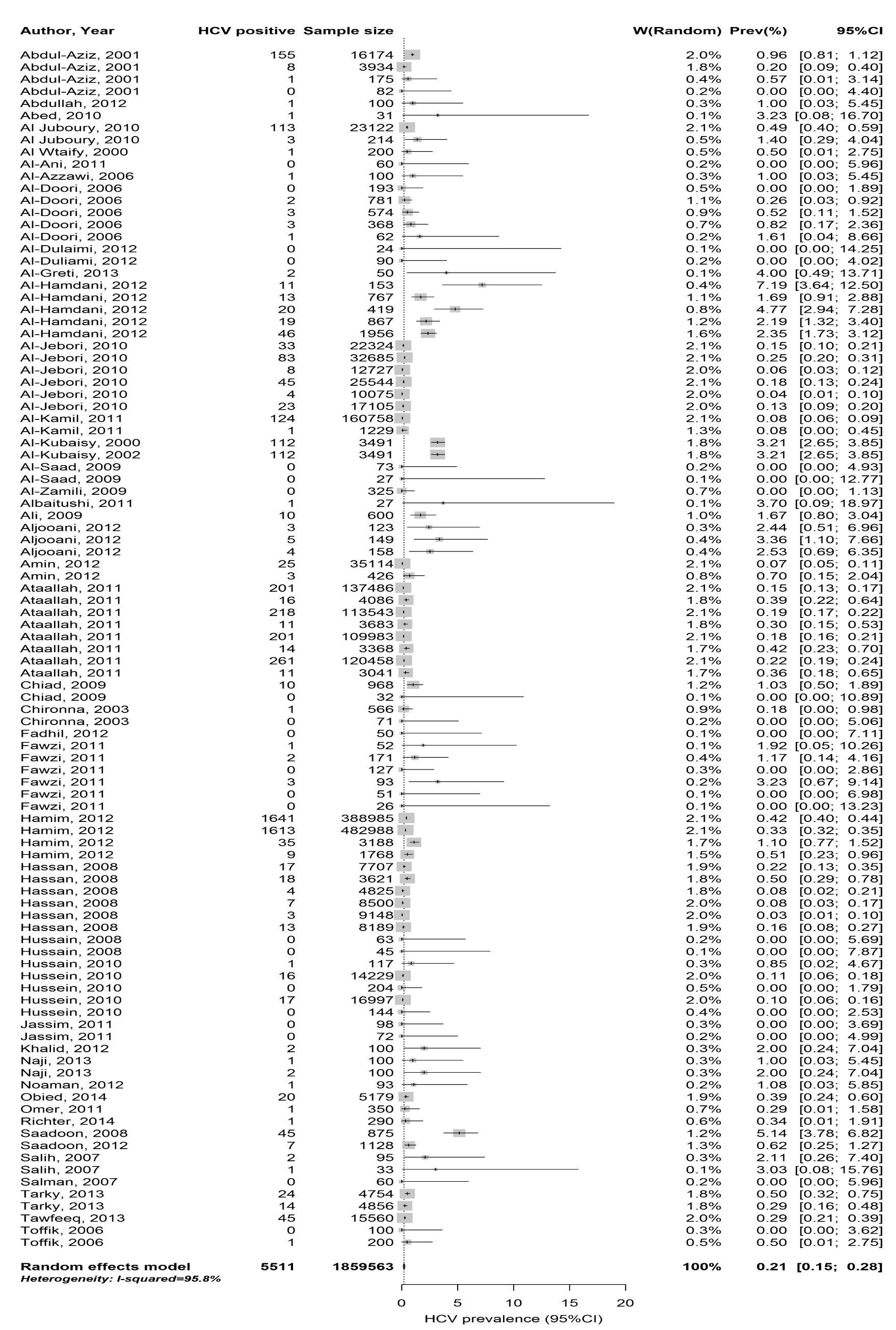

Supplement: S7 Fig — (TIF) [file pone.0135281.s007.tif]

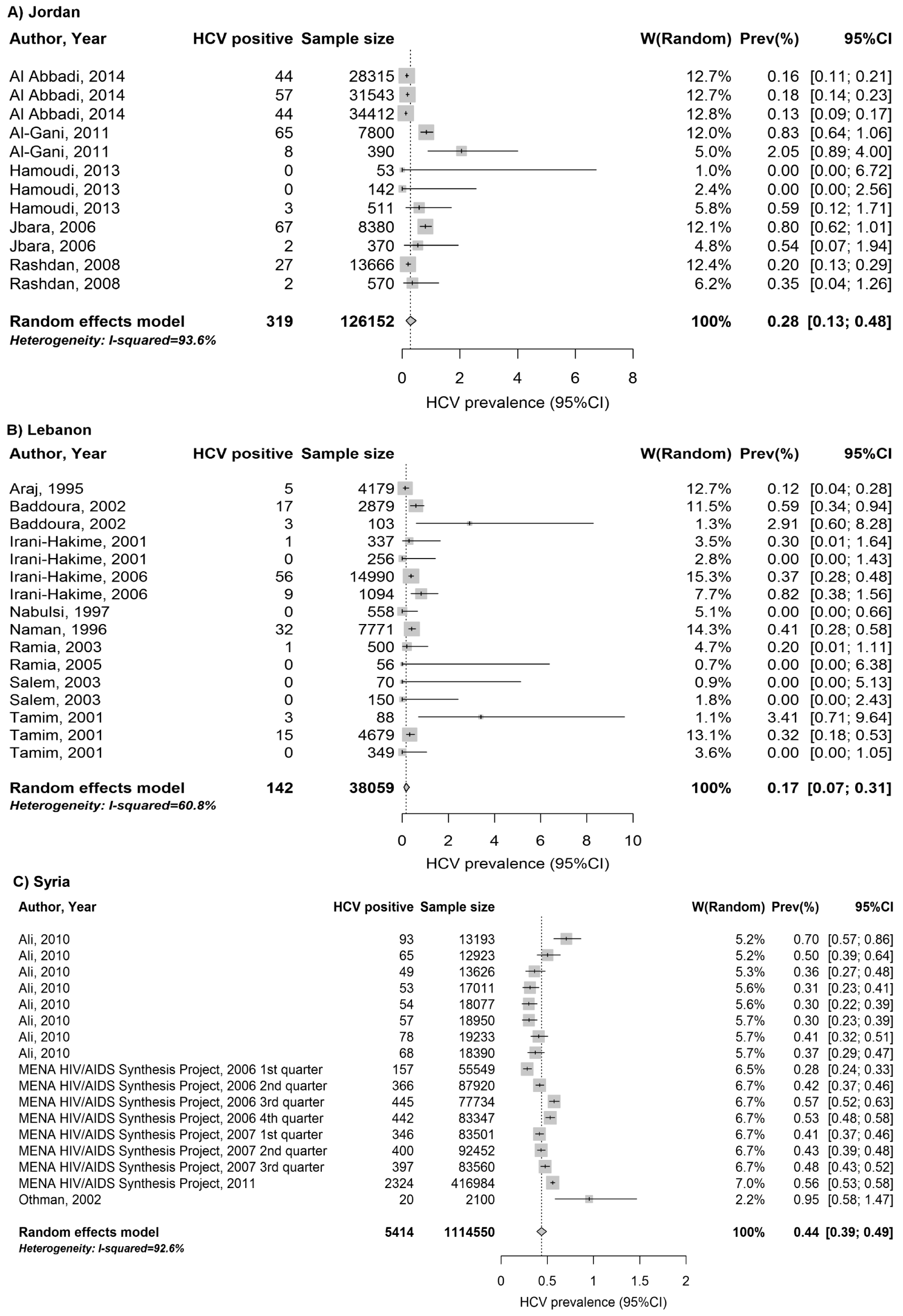

Supplement: S8 Fig — (TIF) [file pone.0135281.s008.tif]

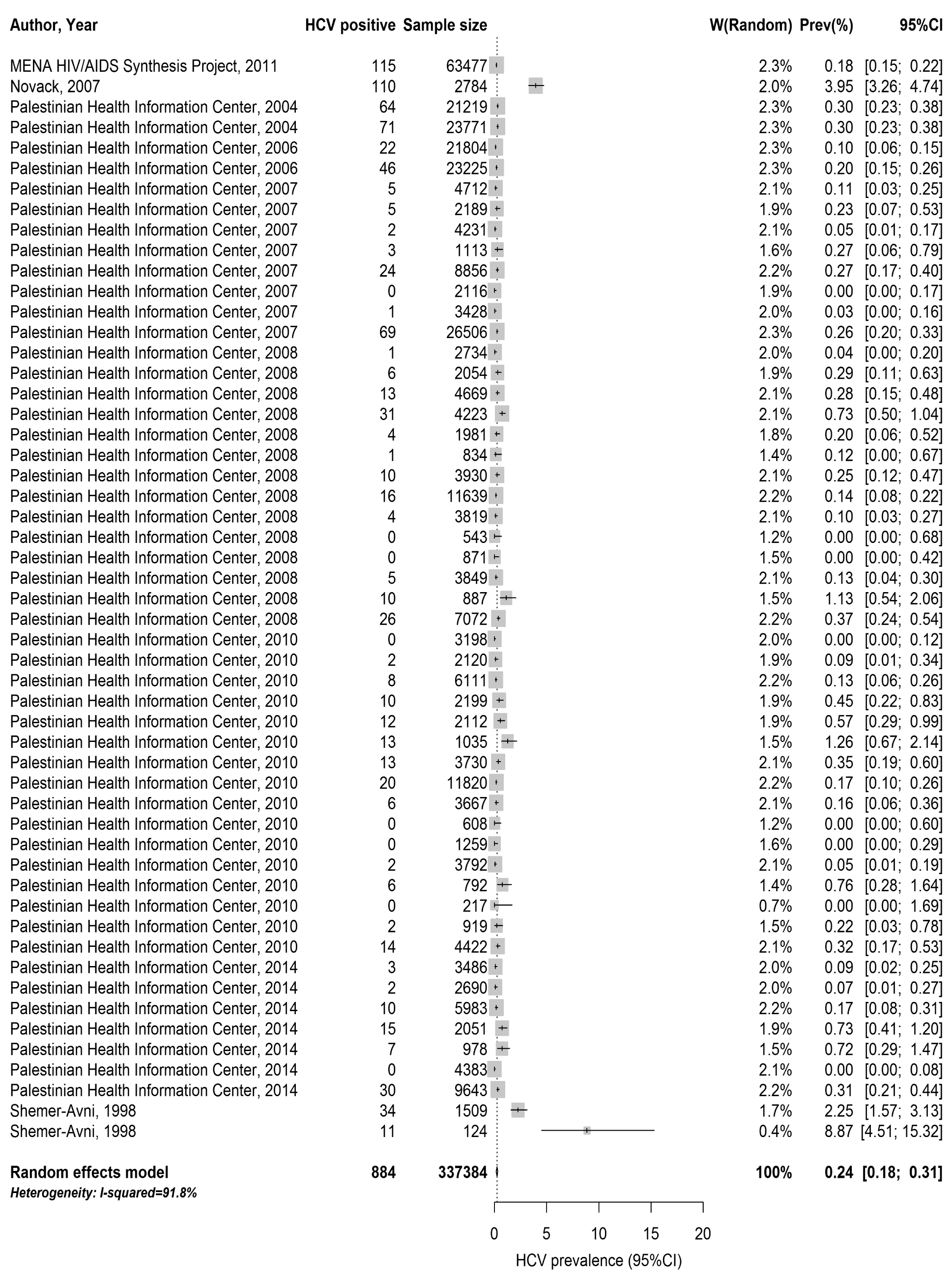

Supplement: S9 Fig — (TIF) [file pone.0135281.s009.tif]
