# Supplementary material for: The Epidemiology of Hepatitis C Virus in the Fertile Crescent: Systematic Review and Meta-Analysis
Source: PLoS One. 2015 Aug 21;10(8):e0135281. doi: 10.1371/journal.pone.0135281 (PMC4546629; doi:10.1371/journal.pone.0135281)
Supplement: S1 Table — (DOCX) [file pone.0135281.s010.docx]

**S1 Table. Lists of variables extracted from relevant reports with hepatitis C virus (HCV) incidence and/or prevalence information or with HCV genotype information.**

| **List of extracted variables from relevant reports with:** | |
| --- | --- |
| HCV incidence and/or prevalence information | HCV genotype information |
| Author, year of publication | Author, year of publication |
| Full citation | Full citation |
| Year of data collection | Year of data collection |
| Publication type | Publication type |
| Country of origin | Country of origin |
| Country of survey | Country of survey |
| City | Study population |
| Study site | Sample size for HCV RNA testing |
| Study design | Sample size of HCV RNA positive individuals |
| Study sampling procedure | Prevalence of HCV genotypes |
| Study population | Prevalence of single and mixed HCV genotypes |
| Characteristics of study population (sex, age, nationality…) | Subtype information |
| Response rate |  |
| Sample size |  |
| HCV incidence (and related follow-up time) |  |
| HCV prevalence |  |
| Type of HCV ascertainment |  |
| Type of assay used for HCV ascertainment |  |
| Type of assay used for confirmatory testing |  |
| HCV RNA prevalence |  |
| Risk factors for HCV infection (identified as significant after controlling for confounders through multivariable regression analyses) |  |
