# Supplementary material for: The Epidemiology of Hepatitis C Virus in the Fertile Crescent: Systematic Review and Meta-Analysis
Source: PLoS One. 2015 Aug 21;10(8):e0135281. doi: 10.1371/journal.pone.0135281 (PMC4546629; doi:10.1371/journal.pone.0135281)
Supplement: S3 Table — (DOCX) [file pone.0135281.s012.docx]

**S3 Table. Studies reporting hepatitis C virus (HCV) RNA prevalence in countries of the Fertile Crescent.**

| **First author, year of publication [citation]** | **Years of data collection** | **Population’s classification based on risk of HCV exposure** | **Population** | **RNA prev among anti-HCV+^*^** | **RNA prev among anti-HCV-^*^** | **RNA prev among whole sample^*^** |
| --- | --- | --- | --- | --- | --- | --- |
| **Iraq (n=12)** |  |  |  |  |  |  |
| Abdullah, 12 [[1](#_ENREF_1)] | 2010 | High risk population | Hemodialysis patients | 26.1% | 3.5% | 12.3% |
| Abdullah, 12 [[2](#_ENREF_2)] | 2005-07 | High risk population | Hemodialysis patients |  |  | 31.3% |
| Al Kubaisy, 06 [[3](#_ENREF_3)] | 1998 | High risk population | Thalassemic patients (2-10 years) | 76% | 16.7% | 61.5% |
| Khaled, 14 [[4](#_ENREF_4)] | 2012 | High risk population | Thalassemic patients | 88% |  |  |
| Shihab, 14 [[5](#_ENREF_5)] | 2012-13 | High risk population | Hemodialysis patients | 61.5% |  |  |
| Abdullah, 12 [[2](#_ENREF_2)] | 2005-07 | Low risk population | Blood donors | 100% |  |  |
| Al-Kubaisy, 02 [[6](#_ENREF_6)] |  | Low risk population | Pregnant women | 62.7% |  |  |
| Chironna, 03 [[7](#_ENREF_7)] |  | Low risk population | Refugees | 100% |  |  |
| Obied, 14 [[8](#_ENREF_8)] | 2012-13 | Low risk population | Blood donors | 65% |  |  |
| Tawfeeq, 13 [[9](#_ENREF_9)] | 2011-12 | Low risk population | Blood donors | 68.9% |  |  |
| Al-Kubaisy, 14 [[10](#_ENREF_10)] | 2000-03 | Special clinical population | Hepatocellular carcinoma patients | 70.8% |  |  |
| Al-Kubaisy, 14 [[10](#_ENREF_10)] | 2000-03 | Special clinical population | Patients with malignant tumors | 22.2% |  |  |
| **Jordan (n=3)** |  |  |  |  |  |  |
| Al-Sweedan, 11 [[11](#_ENREF_11)] | 2008 | High risk population | Thalassemic patients | 50.0% |  |  |
| Bdour, 02 [[12](#_ENREF_12)] |  | High risk population | Hemodialysis patients | 31.5% |  |  |
| Rashdan, 08 [[13](#_ENREF_13)] | 2004-06 | Low risk population | Blood donors | 89.7% |  |  |
| **Lebanon (n=9)** |  |  |  |  |  |  |
| Abdelnour, 97 [[14](#_ENREF_14)] |  | High risk population | Hemodialysis patients | 65% | 10.3% |  |
| Mahfoud, 10 [[15](#_ENREF_15)] | 2007-08 | High risk population | People who inject drugs | 50% |  |  |
| Ramia, 02 [[16](#_ENREF_16)] | 1999-00 | High risk population | Thalassemic patients | 34.5% |  |  |
| Ramia, 03 [[17](#_ENREF_17)] |  | High risk population | Multi-transfused cancer patients | 66.7% |  |  |
| El-Kader, 10 [[18](#_ENREF_18)] | 2007 | High risk population | Hemodialysis patients | 84.1% | 4.9% | 19.1% |
| Irani-Hakime, 01 [[19](#_ENREF_19)] | 1999 | Intermediate risk | Health care workers | 100% |  |  |
| Mahfoud, 10 [[20](#_ENREF_20)] | 2007-08 | Intermediate risk | Prisoners | 50% |  |  |
| Irani-Hakime, 01 [[19](#_ENREF_19)] | 1999 | Low risk population | Blood donors | 100% |  |  |
| Ramia, 03 [[17](#_ENREF_17)] |  | Low risk population | Blood donors | 0% |  |  |
| **Palestine (n=2)** |  |  |  |  |  |  |
| Shemer-Avni, 98 [[21](#_ENREF_21)] |  | Low risk population | Blood donors | 71% |  |  |
| Shemer-Avni, 98 [[21](#_ENREF_21)] |  | Low risk population | Outpatient hospital attendees | 64% |  |  |
| **Syria (n=1)** |  |  |  |  |  |  |
| Abdulkarim, 98 [[22](#_ENREF_22)] |  | High risk population | Hemodialysis patients | 87.5% |  |  |

Prev, prevalence.

^*^The decimal places of the prevalence figures are as reported in the original report, but prevalence figures with more than one decimal places were rounded to one decimal place.

**References**

1. Abdullah AM, Hardan A, Latif II. Genotyping of hepatitis C virus isolates from Iraqi hemodialysis patients by reverse transcription-PCR and one step nested RT-PCR. Diyala Journal of Medicine. 2012;3(1):9-18.

2. Abdullah B. A., Khaled M. D., Maarouf M. N. Detection of hepatitis C virus (HCV) by ELISA, RIBA and Reverse Transcriptase- Polymerase Chain Reaction (RT-PCR) technique among kidney dialysis patients in Nineveh governorate/Iraq. Science Journal of Thi-Qar 2012;3(2):55-67.

3. Al-Kubaisy WA, Al-Naib KT, Habib M. Seroprevalence of hepatitis C virus specific antibodies among Iraqi children with thalassaemia. Eastern Mediterranean health journal = La revue de sante de la Mediterranee orientale = al-Majallah al-sihhiyah li-sharq al-mutawassit. 2006;12(1-2):204-10. Epub 2006/10/14. PubMed PMID: 17037239.

4. Khaled M. D. Prevalence of hepatitis B, hepatitis C and human immunodeficiency virus infection among Thalassemia patients in Ninavha Governorate/Iraq. Journal of Biotechnology Research Center. 2014;8(2):11-3.

5. Shihab SS, Al-Hmudi HA, Al-Edani HS, Mahdi KH. Viral hepatitis infections in Basrah haemodialysis unit: serological diagnosis and viral loading. European Journal of Experimental Biology. 2014;4(2):106-12.

6. Al-Kubaisy WA, Niazi AD, Kubba K. History of miscarriage as a risk factor for hepatitis C virus infection in pregnant Iraqi women. Eastern Mediterranean health journal = La revue de sante de la Mediterranee orientale = al-Majallah al-sihhiyah li-sharq al-mutawassit. 2002;8(2-3):239-44. Epub 2004/09/02. PubMed PMID: 15339110.

7. Chironna M, Germinario C, Lopalco PL, Carrozzini F, Barbuti S, Quarto M. Prevalence rates of viral hepatitis infections in refugee Kurds from Iraq and Turkey. Infection. 2003;31(2):70-4. Epub 2003/04/12. doi: 10.1007/s15010-002-3100-3. PubMed PMID: 12682810.

8. Obied H.M., Alrodhan M.A., Mallah M.O. Molecular and immunological detection of hepatitis C virus infection among blood donors in Al-Muthanna province-Iraq. International Journal of Advanced Research. 2014;2(6):295-315.

9. Tawfeeq WF. Detection of hepatitis C virus infection and genotypes among seropositive blood donors by polymerase chain reaction in babylon Governorate, Iraq. Medical Journal of Babylon. 2013;10(1):25-37.

10. Al-Kubaisy W. A., Obaid K. J., Noor N. M., Ibrahim N. S., Al-Azawi A. A. Hepatitis C virus prevalence and genotyping among hepatocellular carcinoma patients in Baghdad. Asian pacific Journal of Cancer Prevention. 2014;15(18):7725-30.

11. Al-Sweedan SA, Jaradat S, Amer K, Hayajneh W, Haddad H. Seroprevalence and genotyping of hepatitis C virus in multiple transfused Jordanian patients with beta-thalassemia major. [Turkish]. Multipl transfuzyon uygulanan beta-talasemi majorlu urdunlu hastalarda hepatit c virusunun seroprevalansi{dotless} ve genotiplemesi. Turkish Journal of Hematology. 2011;28(1):47-51. doi: <http://dx.doi.org/10.5152/tjh.2011.05>. PubMed PMID: 2011173285.

12. Bdour S. Hepatitis C virus infection in Jordanian haemodialysis units: serological diagnosis and genotyping. Journal of medical microbiology. 2002;51(8):700-4. Epub 2002/08/13. PubMed PMID: 12171303.

13. Rashdan A, Hijjawi S, Jadallah K, Matalka I. Prevalence of hepatitis C virus antibodies among blood donors in Northern Jordan. Jordan Medical Journal. 2008;42(3):179-83. PubMed PMID: 2009005380.

14. Abdelnour GE, Matar GM, Sharara HM, Abdelnoor AM. Detection of anti-hepatitis C-virus antibodies and hepatitis C-virus RNA in Lebanese hemodialysis patients. European journal of epidemiology. 1997;13(8):863-7. Epub 1998/02/26. PubMed PMID: 9476813.

15. Mahfoud Z, Kassak K, Kreidieh K, Shamra S, Ramia S. Distribution of hepatitis C virus genotypes among injecting drug users in Lebanon. Virology journal. 2010;7:96. Epub 2010/05/15. doi: 10.1186/1743-422x-7-96. PubMed PMID: 20465784; PubMed Central PMCID: PMC2885342.

16. Ramia S, Koussa S, Taher A, Haraki S, Klayme S, Sarkis D, et al. Hepatitis-C-virus genotypes and hepatitis-G-virus infection in Lebanese thalassaemics. Annals of tropical medicine and parasitology. 2002;96(2):197-202. Epub 2002/06/26. doi: 10.1179/000349802125000439. PubMed PMID: 12080981.

17. Ramia S, Klayme S, Naman R. Infection with hepatitis B and C viruses and human retroviruses (HTLV-I and HIV) among high-risk Lebanese patients. Annals of tropical medicine and parasitology. 2003;97(2):187-92. Epub 2003/06/14. doi: 10.1179/000349803235001363. PubMed PMID: 12803874.

18. El-kader YE-OA, Elmanama AA, Ayesh BM. Prevalence and risk factors of hepatitis B and C viruses among haemodialysis patients in Gaza strip, Palestine. Virology journal. 2010;7:210. Epub 2010/09/03. doi: 10.1186/1743-422x-7-210. PubMed PMID: 20809985; PubMed Central PMCID: PMC2942824.

19. Irani-Hakime N, Aoun J, Khoury S, Samaha HR, Tamim H, Almawi WY. Seroprevalence of hepatitis C infection among health care personnel in Beirut, Lebanon. American journal of infection control. 2001;29(1):20-3. Epub 2001/02/15. PubMed PMID: 11172314.

20. Mahfoud Z, Kassak K, Kreidieh K, Shamra S, Ramia S. Prevalence of antibodies to human immunodeficiency virus (HIV), hepatitis B and hepatitis C and risk factors in prisoners in Lebanon. Journal of infection in developing countries. 2010;4(3):144-9. Epub 2010/03/31. PubMed PMID: 20351454.

21. Shemer-Avni Y, el Astal Z, Kemper O, el Najjar KJ, Yaari A, Hanuka N, et al. Hepatitis C virus infection and genotypes in Southern Israel and the Gaza Strip. Journal of medical virology. 1998;56(3):230-3. Epub 1998/10/23. PubMed PMID: 9783690.

22. Abdulkarim AS, Zein NN, Germer JJ, Kolbert CP, Kabbani L, Krajnik KL, et al. Hepatitis C virus genotypes and hepatitis G virus in hemodialysis patients from Syria: identification of two novel hepatitis C virus subtypes. The American journal of tropical medicine and hygiene. 1998;59(4):571-6. Epub 1998/10/28. PubMed PMID: 9790432.
