# Supplementary material for: The Epidemiology of Hepatitis C Virus in the Fertile Crescent: Systematic Review and Meta-Analysis
Source: PLoS One. 2015 Aug 21;10(8):e0135281. doi: 10.1371/journal.pone.0135281 (PMC4546629; doi:10.1371/journal.pone.0135281)
Supplement: S4 Table — (DOCX) [file pone.0135281.s013.docx]

**S4 Table.** **Summary of precision and risk of bias assessment for hepatitis C virus (HCV) incidence and prevalence measures extracted from eligible reports.**

| **Quality assessment** | **HCV incidence measures** | | **HCV prevalence measures** | |
| --- | --- | --- | --- | --- |
|  | **n** | **%** | **n** | **%** |
| **Precision of estimates** |  |  |  |  |
| High precision | 2 | 25.0 | 165 | 81.3 |
| Low precision | 6 | 75.0 | 38 | 18.7 |
| **Risk of bias quality domains** |  |  |  |  |
| **Sampling methodology** |  |  |  |  |
| Low risk of bias | 0 | 0.0 | 26 | 14.1 |
| High risk of bias | 8 | 100.0 | 158 | 85.9 |
| **HCV ascertainment** |  |  |  |  |
| Low risk of bias | 7 | 87.5 | 181 | 98.4 |
| High risk of bias | 0 | 0 | 3 | 1.6 |
| Unclear^*^ | 1 | 12.5 | 0 | 0 |
| **Response rate** |  |  |  |  |
| Low risk of bias | 6 | 75.0 | 92 | 50.0 |
| High risk of bias | 1 | 12.5 | 7 | 3.8 |
| Unclear^*^ | 1 | 12.5 | 85 | 46.2 |
| **Total number of studies where risk of bias assessment was possible** | **8** | **100.0** | **184** | **90.6** |
| **Unknown**^**^ |  |  | 19 | 9.4 |
| **Total number of studies** | **8** | **100.0** | **203**^†^ | **100.0** |
| **Summary of risk of bias assessment for HCV incidence and prevalence measures** | | | | |
|  | **n** | **%** |  |  |
| **Low risk of bias** |  |  |  |  |
| In at least one quality domain | 190 | 99.0 |  |  |
| In at least two quality domains | 92 | 47.9 |  |  |
| In all three quality domains | 15 | 7.8 |  |  |
| **High risk of bias** |  |  |  |  |
| In at least one quality domain | 168 | 87.5 |  |  |
| In at least two quality domains | 7 | 3.6 |  |  |
| In all three quality domains | 1 | 0.5 |  |  |
| **Total number of studies where risk of bias assessment was possible** | **192** | **91.0** |  |  |
| **Total number of studies** | **211** | **100.0** |  |  |

^*^Studies with missing information for any of the domains were classified as having unclear ROB for that specific domain.

^**^Studies extracted from regional databases with limited description of the sample not permitting the conduct of ROB assessment were classified as being of unknown quality.

^†^The ROB assessment was performed for all HCV prevalence measures reported in Tables 2 and 3 in main article and S2 Table. This assessment excludes 37 studies where sample size was less than 50 participants.
