# Supplementary material for: The Epidemiology of Hepatitis C Virus in the Fertile Crescent: Systematic Review and Meta-Analysis
Source: PLoS One. 2015 Aug 21;10(8):e0135281. doi: 10.1371/journal.pone.0135281 (PMC4546629; doi:10.1371/journal.pone.0135281)
Supplement: S5 Table — (DOCX) [file pone.0135281.s014.docx]

**S5 Table.** **Precision and risk of bias (ROB) assessment for individual hepatitis C virus (HCV) incidence measures in countries of the Fertile Crescent.**

| **First author, year of publication [citation]** | **Years of data collection** | **Population** | **Sample size** | **HCV sero-conversion risk** | **Precision** | **Study sampling procedure** | **HCV ascertain-ment** | **Response rate** |
| --- | --- | --- | --- | --- | --- | --- | --- | --- |
| **Jordan** |  |  |  |  |  |  |  |  |
| Batieha, 07 [[1](#_ENREF_1)] | 2003 | Hemodialysis patients | 1300 | 9.2% | High | High ROB | Unclear | Low ROB |
| **Iraq** |  |  |  |  |  |  |  |  |
| Al-Rubaie, 11 [[2](#_ENREF_2)] | 2009 | Hemodialysis patients | 57 | 40.3% | Low | High ROB | Low ROB | Unclear^*^ |
| Al-Jadiry, 08 [[3](#_ENREF_3)] | 2007 | Pediatric patients with acute lymphoblastic leukemia | 123 | 3.2% | High | High ROB | Low ROB | Low ROB |
| Al-Kubaisy, 00 [[4](#_ENREF_4)] |  | Newborns to HCV infected women | 26 | 0% | Low | High ROB | Low ROB | Low ROB |
| Al-Ali, 14 [[5](#_ENREF_5)] | 2006-07 | Pediatric cancer patients on chemotherapy | 85 | 3.2% | Low | High ROB | Low ROB | High ROB |
| Al-Ani, 11 [[6](#_ENREF_6)] | 2007-09 | Healthy children | 60 | 0% | Low | High ROB | Low ROB | Low ROB |
| Al-Ani, 11 [[6](#_ENREF_6)] | 2007-09 | Pediatric patients with leukemia on chemotherapy | 29 | 3.5% | Low | High ROB | Low ROB | Low ROB |
| Al-Ani, 11 [[6](#_ENREF_6)] | 2007-09 | Pediatric patients with leukemia who have had their baseline screening prior to chemotherapy | 27 | 0% | Low | High ROB | Low ROB | Low ROB |

^*^Studies with missing information for any of the domains were classified as having unclear ROB for that specific domain.

**References**

1. Batieha A, Abdallah S, Maghaireh M, Awad Z, Al-Akash N, Batieneh A, et al. Epidemiology and cost of haemodialysis in Jordan. Eastern Mediterranean Health Journal. 2007;13(3):654-63. PubMed PMID: 2007356348.

2. Al-Rubaie H. MH., Malik A.S. Seroconversion rate of hepatitis C virus infection among haemodialysis patients in Al-Kadhimiya Teaching Hospital (dialysis unit). Iraqi Journal of Medical Sciences. 2011;9(4):343-9.

3. Al-Jadiry M. Viral hepatitis markers screen in children with acute lymphoblastic leukemia experience of Children Welfare Teaching Hospital. Journal of the Faculty of Medicine of Baghdad. 2008;50(2):223-30.

4. Al-Kubaisy W. A., Niazi A., Kubba K. Lack of mother-to-newborn transmission of hepatitis C virus in Iraqi women: a prospective study with hepatitis C virus RNA testing. Journal of the Arab Board of Health Specializations. 2000;2(2).

5. Al-Ali N. AA., AL-Kayatt T. N. Seroprevalence of hepatitis B & C in pediatric malignancies. Iraqi Postgraduate Medical Journal. 2014;13(2):262-7.

6. Al-Ani M. H., Rasul T. H. Hepatitis B and C viral infections in children with acute leukemia in Erbil city. Journal of the Arab Board of Health Specializations. 2011;12(1):21-9.
