# Supplementary material for: The Epidemiology of Hepatitis C Virus in the Fertile Crescent: Systematic Review and Meta-Analysis
Source: PLoS One. 2015 Aug 21;10(8):e0135281. doi: 10.1371/journal.pone.0135281 (PMC4546629; doi:10.1371/journal.pone.0135281)
Supplement: S6 Table — (DOCX) [file pone.0135281.s015.docx]

**S6 Table.** **Precision and risk of bias (ROB) assessment for individual hepatitis C virus (HCV) prevalence measures among populations at high risk in countries of the Fertile Crescent.**

| **First author, year of publication [citation]** | **Years of data collection** | **Population** | **Sample size** | **HCV prev** | **Preci-sion** | **Study sampling procedure** | **HCV ascertain-ment** | **Response rate** |
| --- | --- | --- | --- | --- | --- | --- | --- | --- |
| **Iraq (n=36)** |  |  |  |  |  |  |  |  |
| Abdul-Aziz, 01 [[1](#_ENREF_1)] | 1999-01 | Hemodialysis patients | 95 | 0% | Low | High ROB | Low ROB | Low ROB |
| Abdul-Aziz, 01 [[1](#_ENREF_1)] | 1999-01 | Thalassemic patients | 163 | 8% | High | High ROB | Low ROB | Low ROB |
| Abdul-Karim, 11 [[2](#_ENREF_2)] | 2008 | Patients with bleeding disorders | 243 | 40.3% | High | High ROB | Low ROB | High ROB |
| Abdullah, 12 [[3](#_ENREF_3)] | 2010 | Hemodialysis patients | 236 | 39% | High | High ROB | Low ROB | Unclear^*^ |
| Abdullah, 12 [[4](#_ENREF_4)] | 2005-07 | Hemodialysis patients | 80 | 28.7% | Low | High ROB | Low ROB | Unclear^*^ |
| Abed, 10 [[5](#_ENREF_5)] | 2008 | Thalassemic patients | 111 | 46% | High | High ROB | Low ROB | Unclear^*^ |
| Albahadle, 13 [[6](#_ENREF_6)] | 2011 | Thalassemic patients (0-18 years) | 206 | 19.9% | High | High ROB | Low ROB | Low ROB |
| Al-Barzinji, 06 [[7](#_ENREF_7)] | 2004 | Multi-transfused leukemia patients on chemotherapy (1-12 years) | 88 | 4.5% | Low | High ROB | Low ROB | Unclear^*^ |
| Al-Beldawi, 10 [[8](#_ENREF_8)] | 2006 | Hemophilia patients (<20 years) | 200 | 40% | High | High ROB | Low ROB | Low ROB |
| Al-Dulaimi, 12 [[9](#_ENREF_9)] | 2010-11 | Hemodialysis patients | 84 | 14.3% | Low | High ROB | Low ROB | Unclear^*^ |
| Al-Greti, 13 [[10](#_ENREF_10)] | 2011-12 | Thalassemic patients | 100 | 37% | High | High ROB | Low ROB | Unclear^*^ |
| Al-Juboori, 12 [[11](#_ENREF_11)] | 2010-11 | Thalassemic patients (<20 years) | 50 | 10% | Low | High ROB | Low ROB | Unclear^*^ |
| Al Kubaisy, 06 [[12](#_ENREF_12)] | 1998 | Thalassemic patients (2-10 years) | 559 | 67.3% | High | Low ROB | Low ROB | Unclear^*^ |
| Al-Marzoqi, 09 [[13](#_ENREF_13)] | 2008 | Thalassemic patients (children) | 50 | 38% | Low | High ROB | Low ROB | Unclear^*^ |
| Al-Mashhadani, 07 [[14](#_ENREF_14)] | 2002 | Hemodialysis patients | 87 | 11.5% | Low | High ROB | Low ROB | Unclear^*^ |
| Al-Thwani, 06 [[15](#_ENREF_15)] | 1997-04 | Hemophilia patients | 100 | 25% | High | High ROB | Low ROB | Unclear^*^ |
| Al Wtaify, 00 [[16](#_ENREF_16)] | 1998-99 | Thalassemic patients (children) | 200 | 9.5% | High | High ROB | Low ROB | Unclear^*^ |
| Al-Zamili, 09 [[17](#_ENREF_17)] | 2007-08 | Thalassemic patients (<20 years) | 325 | 4% | High | High ROB | Low ROB | Unclear^*^ |
| Easa, 09 [[18](#_ENREF_18)] | 2007-08 | Thalassemic patients (5-18 years) | 140 | 26.4% | High | High ROB | Low ROB | Unclear^*^ |
| Fadhil, 12 [[19](#_ENREF_19)] | 2010-11 | Thalassemic patients | 200 | 21% | High | High ROB | Low ROB | Unclear^*^ |
| Hashem, 13 [[20](#_ENREF_20)] | 2011 | Thalassemic patients | 284 | 19% | High | High ROB | Low ROB | Low ROB |
| Hassan, 08 [[21](#_ENREF_21)] | 1996-01 | Thalassemic patients | 136 | 16.9% | High | High ROB | Low ROB | Low ROB |
| Khaled, 14 [[22](#_ENREF_22)] | 2012 | Thalassemic patients | 480 | 10.4% | High | High ROB | Low ROB | Low ROB |
| Khalid, 12 [[23](#_ENREF_23)] |  | Thalassemic patients | 200 | 17.5% | High | High ROB | Low ROB | Unclear^*^ |
| Khattab, 08 [[24](#_ENREF_24)] | 2003-05 | Hemodialysis patients | 169 | 7.1% | High | High ROB | Low ROB | Unclear^*^ |
| Khattab, 10 [[25](#_ENREF_25)] | 2003-08 | Hemodialysis patients | 244 | 4.9% | High | High ROB | Low ROB | Unclear^*^ |
| Mnuti, 11 [[26](#_ENREF_26)] | 2008-10 | Hemodialysis patients | 100 | 41% | High | High ROB | Low ROB | Unclear^*^ |
| Muhsin, 13 [[27](#_ENREF_27)] | 2011-12 | Hemophilia patients | 60 | 6.7% | Low | Low ROB | Low ROB | Unclear^*^ |
| Muhsin, 13 [[27](#_ENREF_27)] | 2011-12 | Thalassemic patients | 56 | 25% | Low | Low ROB | Low ROB | Unclear^*^ |
| Mustafa, 10 [[28](#_ENREF_28)] | 2003-04 | Thalassemic patients (<20 years) | 626 | 28.1% | High | High ROB | Low ROB | Low ROB |
| Omer, 2011 [[29](#_ENREF_29)] | 2006-08 | Multi-transfused leukemia patients | 291 | 3.4 | High | High ROB | Low ROB | Unclear^*^ |
| Omer, 11 [[30](#_ENREF_30)] | 2010 | Thalassemic patients | 54 | 7.4% | Low | High ROB | Low ROB | Low ROB |
| Raham, 11 [[31](#_ENREF_31)] | 1999-00 | Thalassemic patients | 110 | 26.4% | High | High ROB | Low ROB | High ROB |
| Ramzi, 10 [[32](#_ENREF_32)] | 2009 | Hemodialysis patients | 101 | 26.7% | High | High ROB | Low ROB | Unclear^*^ |
| Saadoon, 12 [[33](#_ENREF_33)] |  | Thalassemic patients | 162 | 34.6% | High | High ROB | Low ROB | Low ROB |
| Shihab, 14 [[34](#_ENREF_34)] | 2012-13 | Hemodialysis patients | 122 | 42.6% | High | High ROB | Low ROB | Unclear^*^ |
| **Jordan (n=7)** |  |  |  |  |  |  |  |  |
| Al-Jamal, 09 [[35](#_ENREF_35)] | 2007-08 | Hemodialysis patients | 120 | 28% | High | High ROB | Low ROB | Unclear^*^ |
| Al-Sweedan, 11 [[36](#_ENREF_36)] | 2008 | Thalassemic patients | 122 | 32.8% | High | High ROB | Low ROB | Unclear^*^ |
| Batchoun, 11 [[37](#_ENREF_37)] |  | Hemodialysis patients | 134 | 47.7% | High | High ROB | Low ROB | Unclear^*^ |
| Batieha, 07 [[38](#_ENREF_38)] | 2003 | Hemodialysis patients | 1711 | 21% | High | High ROB | Low ROB | Low ROB |
| Bdour, 02 [[39](#_ENREF_39)] |  | Hemodialysis patients | 283 | 32.5% | High | High ROB | Low ROB | Unclear^*^ |
| Ghunaimat, 07 [[40](#_ENREF_40)] |  | Hemodialysis patients | 209 | 49.8% | High | High ROB | Low ROB | Unclear^*^ |
| Said, 95 [[41](#_ENREF_41)] | 1994 | Hemodialysis patients | 273 | 24.5% | High | High ROB | Low ROB | Unclear^*^ |
| **Lebanon (n=6)** |  |  |  |  |  |  |  |  |
| Abdelnour, 97 [[42](#_ENREF_42)] |  | Hemodialysis patients | 108 | 16% | High | High ROB | Low ROB | Unclear^*^ |
| Inati, 09 [[43](#_ENREF_43)] |  | Thalassemic patients | 200 | 0% | High | Low ROB | Low ROB | Unclear^*^ |
| Mahfoud, 10 [[44](#_ENREF_44)] | 2007-08 | People who inject drugs | 106 | 52.8% | High | Low ROB | Low ROB | Low ROB |
| Naman, 96 [[45](#_ENREF_45)] |  | Hemodialysis patients | 317 | 27% | High | High ROB | Low ROB | Unclear^*^ |
| Ramia, 02 [[46](#_ENREF_46)] | 1999-00 | Thalassemic patients | 395 | 14.0% | High | High ROB | Low ROB | Low ROB |
| Ramia, 03 [[47](#_ENREF_47)] |  | Multi-transfused cancer patients | 65 | 4.6% | Low | High ROB | Low ROB | Unclear^*^ |
| **Palestine (n=3)** |  |  |  |  |  |  |  |  |
| Dumaidi, 14 [[48](#_ENREF_48)] | 2012-13 | Hemodialysis patients | 146 | 27.4% | High | High ROB | Low ROB | Low ROB |
| El-Kader, 10 [[49](#_ENREF_49)] | 2007 | Hemodialysis patients | 246 | 17.9% | High | High ROB | Low ROB | Unclear^*^ |
| Stulhofer, 12 [[50](#_ENREF_50)] | 2010 | People who inject drugs | 199 | 45.2% | High | Low ROB | Low ROB | Low ROB |
| **Syria (n=5)** |  |  |  |  |  |  |  |  |
| Abdulkarim, 98 [[51](#_ENREF_51)] |  | Hemodialysis patients | 120 | 75% | High | High ROB | Low ROB | Unclear^*^ |
| Ali, 12 [[52](#_ENREF_52)] | 2007-11 | Hemophilia patients | 375 | 20.5% | High | High ROB | Low ROB | High ROB |
| Syrian MOH, 08 [[53](#_ENREF_53)] | 2006 | People who inject drugs | 336 | 21% | Low | High ROB | High ROB | High ROB |
| Moukeh, 09 [[54](#_ENREF_54)] | 2006 | Hemodialysis patients | 550 | 54.4% | High | High ROB | Low ROB | Low ROB |
| Othman, 01 [[55](#_ENREF_55)] | 1996 | Hemodialysis patients | 139 | 48.9% | High | High ROB | Low ROB | High ROB |

MENA HIV/AIDS ESP, Middle East and North Africa HIV/AIDS Epidemiology Synthesis Project database; MOH, Ministry of Health; Prev: prevalence.

^*^Studies with missing information for any of the domains were classified as having unclear ROB for that specific domain.

**References**

1. Abdul-Aziz M, Abdul-Karem K, Shamse-El-Den S, Al-Moula GA. Prevalence of hepatitis B & C among people attending Kirkuk Public Health Laboratory. Al-Taqani. 2001;23(3):6-15.

2. Abdul-Karim E. T. The prevalence of hepatitis in patients with congenital coagulation disorders. Journal of the Arab Board of Health Specializations. 2011;12(3):9-14.

3. Abdullah AM, Hardan A, Latif II. Genotyping of hepatitis C virus isolates from Iraqi hemodialysis patients by reverse transcription-PCR and one step nested RT-PCR. Diyala Journal of Medicine. 2012;3(1):9-18.

4. Abdullah B. A., Khaled M. D., Maarouf M. N. Detection of hepatitis C virus (HCV) by ELISA, RIBA and Reverse Transcriptase- Polymerase Chain Reaction (RT-PCR) technique among kidney dialysis patients in Nineveh governorate/Iraq. Science Journal of Thi-Qar 2012;3(2):55-67.

5. Abed B. A. Prevalence of hepatitis C virus (HCV) among thalassemia patients in Ibn Al-Balady Hospital. Journal of Al-Nahrain University. 2010;13(1):121-6.

6. Albahadle AK. J., Abdul-Abass A., Ali A. H. Prevalence of hepatitis C infection among mutlitransfused thalassemia major patients in Ibn-AlBalady center for thalassemia. Al-Qadisiah Medical Journal. 2013;9(15):73-84.

7. Al-Barzinji R M. Patients with childhood leukemia are at high risk for transfusion-transmitted HBV and HCV infections. The Iraqi Postgraduate Medical Journal. 2006;5(4):442-6.

8. Al-Beldawi D. H. The risk factors of inhibitor development and hepatitis C virus among hemophilic patients in Children Welfare Teaching Hospital. Journal of the Faculty of Medicine of Baghdad. 2010;52(1):4-8.

9. Al-Dulaimi S. B. K., Al-Ubadi A. E., Al-Ubadi A. E., Al-Bayatti E. N., Al-Saday S. D. K. Toxoplasma gondii, HCV, and HBV seroprevalence in Haemodialysis patients with chronic renal failure in Al-Kindy Hospital Baghdad, Iraqi. Al-Mustansiriyah Journal of Science. 2012;23(5):33-8.

10. Al-Greti S. H. H. Prevalence of hepatitis C virus in beta-thalassemia major patients at Karbala governorate. Journal of Babylon University for Pure and Applied Sciences. 2013;21(8):2801-5.

11. Al-Juboori L.F. Hepatitis C virus in thalassemia patients in Tikrit. Medical Journal of Tikrit. 2012;1(181):95-100.

12. Al-Kubaisy WA, Al-Naib KT, Habib M. Seroprevalence of hepatitis C virus specific antibodies among Iraqi children with thalassaemia. Eastern Mediterranean health journal = La revue de sante de la Mediterranee orientale = al-Majallah al-sihhiyah li-sharq al-mutawassit. 2006;12(1-2):204-10. Epub 2006/10/14. PubMed PMID: 17037239.

13. Al-Marzoqi A. H., Shemmran A. R., Al-Hindi Z., Al-Taee Z. M., Al-Nafee' M. K. Bacterial and viral infections associated with thalassemia in Hillah city. Journal of Al-Qadisiyah for Pure Science. 2009;14(3):1-15.

14. Al-Mashhadani JI. Hepatitis C virus infection among haemodialysis patients in Al-Anbar governorate. Iraqi Journal of Community Medicine. 2007;20(1):20-3.

15. Al-Thwani A. N., Al-Yassiri A. S. Using ELISA & ELFA for diagnosis of infection with hepatitis C, B and HIV viruses among Iraqi haemophilic patients. Iraqi Journal of Biotechnology. 2006;5(1):70-84.

16. Al Wtaify AS, Hassan MK. Prevalence of hepatitis C virus infection among multitransfused thalassemic children in Basrah - Iraq. Qatar Medical Journal. 2000;9(2):58-61. PubMed PMID: 2001134222.

17. Al-Zamili A. H., Al-Shebani A. M., Mohsin R. K. Prevalence of hepatitis C viral infection among multi-transfused thalassemic patients. Al-Qadisiah Medical Journal. 2009;5(7):89-99.

18. Easa Z. O. Complications of high serum ferritin level after splenectomy in B thalassemic patients. Kufa Medical Journal. 2009;12(1):243-50.

19. Fadhil R. S., Al-Khalidi S. J., Hussien S. M., Hasoon H. A., Hussien R. A., Alwan L. H. Complications of HBV, HCV and HIV infections in patients with Coolyze Anemia in Baghdad. Iraqi Journal of Cancer and Medical Genetics. 2012;5(1):59-63.

20. Hashem N. A., Abdel-Rida Z. A., Taan G. M., Abdel-Hussein A. Spread of viral hepatitis among thalassemic patients in Maysan province. Al-Mustansiriyah Journal of Science. 2013;24(3):51-6.

21. Hassan A. S. Prevalence of anti-hepatitis C virus antibodies among blood donors and risky groups in Diyala. Journal of the Faculty of Medicine of Baghdad. 2008;50(4):467-70.

22. Khaled M. D. Prevalence of hepatitis B, hepatitis C and human immunodeficiency virus infection among Thalassemia patients in Ninavha Governorate/Iraq. Journal of Biotechnology Research Center. 2014;8(2):11-3.

23. Khalid MD, Abdullah BA. Prevalence of anti-HCV antibodies among thalassemia patients in Mosul City, Iraq. Journal of Life Sciences. 2012;6(5):489-91.

24. Khattab OS. Prevalence and risk factors for hepatitis C virus infection in hemodialysis patients in an Iraqi renal transplant center. Saudi journal of kidney diseases and transplantation : an official publication of the Saudi Center for Organ Transplantation, Saudi Arabia. 2008;19(1):110-5. Epub 2007/12/19. PubMed PMID: 18087139.

25. Khattab O. S. How to decrease the prevalence of hepatitis C in Iraqi hemodialysis patients. The Iraqi Postgraduate Medical Journal. 2010;9(1):31-5.

26. Mnuti J. K., Al-Abbudi F. A. Hepatitis C virus infection assessment among chronic hemodialysis patients in Al-Kadhmiya Teaching Hospital. Iraqi Postgraduate Medical Journal. 2011;10(4):460-4.

27. Muhsin M. A., Abdul-Husin I. F. Seroprevalence of hepatitis B and C among thalassemic, haemophilic patients in Babylon Governorate-Iraq. Medical Journal of Babylon. 2013;10(2):445-54.

28. Mustafa B. The prevalence of hepatitis- B and C serological markers among patients with thalassemia in Mosul. Iraqi Journal of Pharmacy. 2010;9 & 10(1):66-9.

29. Omer AR, Salih JI, Al-Nakshabandi AA. Frequency of blood-borne viral infections among leukemic patients in central Iraq. Saudi medical journal. 2011;32(1):55-61. Epub 2011/01/08. PubMed PMID: 21212918.

30. Omer W. A., Jamaleldin Z. A. Evaluation of some health and social parameters in Beta-thalassemia major patients in Ninawa governorate. Iraqi Medical Journal. 2011;57(2):106-10.

31. Raham TF, Abdul Wahed SS, Alhaddad H. N. Prevalence of hepatitis C among patients with Bthalassemia in Diyala-IRAQ. Al-Taqani. 2011;24(4):113-20.

32. Ramzi Z. S., Abdulla A. A., Al-Hadithi T., Al-Tawil N. Prevalence and risk factors for hepatitis C virus infection in hemodialysis patients in Sulaimani. Zanco Journal of Medical Sciences. 2010;14(1):44-50.

33. Saadoon A. A. Prevalence of viral hepatitis B and C among selected group in Thi-Qar. Thi-Qar Medical Journal. 2012;6(1):79-89.

34. Shihab SS, Al-Hmudi HA, Al-Edani HS, Mahdi KH. Viral hepatitis infections in Basrah haemodialysis unit: serological diagnosis and viral loading. European Journal of Experimental Biology. 2014;4(2):106-12.

35. Al-Jamal M, Al-Qudah A, Al-Shishi KF, Al-Sarayreh A, Al-Quraan L. Hepatitis C virus (HCV) infection in hemodialysis patients in the south of Jordan. Saudi journal of kidney diseases and transplantation : an official publication of the Saudi Center for Organ Transplantation, Saudi Arabia. 2009;20(3):488-92. Epub 2009/05/06. PubMed PMID: 19414962.

36. Al-Sweedan SA, Jaradat S, Amer K, Hayajneh W, Haddad H. Seroprevalence and genotyping of hepatitis C virus in multiple transfused Jordanian patients with beta-thalassemia major. [Turkish]. Multipl transfuzyon uygulanan beta-talasemi majorlu urdunlu hastalarda hepatit c virusunun seroprevalansi{dotless} ve genotiplemesi. Turkish Journal of Hematology. 2011;28(1):47-51. doi: <http://dx.doi.org/10.5152/tjh.2011.05>. PubMed PMID: 2011173285.

37. Batchoun RG, Al-Najdawi MA, Al-Taamary S. Anti-ENA antibody profile in hepatitis C patients undergoing hemodialysis. Saudi journal of kidney diseases and transplantation : an official publication of the Saudi Center for Organ Transplantation, Saudi Arabia. 2011;22(4):682-8. Epub 2011/07/12. PubMed PMID: 21743211.

38. Batieha A, Abdallah S, Maghaireh M, Awad Z, Al-Akash N, Batieneh A, et al. Epidemiology and cost of haemodialysis in Jordan. Eastern Mediterranean Health Journal. 2007;13(3):654-63. PubMed PMID: 2007356348.

39. Bdour S. Hepatitis C virus infection in Jordanian haemodialysis units: serological diagnosis and genotyping. Journal of medical microbiology. 2002;51(8):700-4. Epub 2002/08/13. PubMed PMID: 12171303.

40. Ghunaimat M., Al-Mrayat Z., Abbadi R., Akash N. Point prevalence of hepatitis C antibodies among hemodialysis patients at king Hussein Medical Center. Journal of the Royal Medical Services. 2007;14(2):63-7.

41. Said RA, Hamzeh YY, Mehyar NS, Rababah MS. Hepatitis C virus infection in hemodialysis patients in jordan. Saudi journal of kidney diseases and transplantation : an official publication of the Saudi Center for Organ Transplantation, Saudi Arabia. 1995;6(2):140-3. Epub 1995/04/01. PubMed PMID: 18583853.

42. Abdelnour GE, Matar GM, Sharara HM, Abdelnoor AM. Detection of anti-hepatitis C-virus antibodies and hepatitis C-virus RNA in Lebanese hemodialysis patients. European journal of epidemiology. 1997;13(8):863-7. Epub 1998/02/26. PubMed PMID: 9476813.

43. Inati A, Musallam K, Taha M, Ziade F, Taher A. Magnetic resonance imaging T2* in the evaluation of cardiac Iron overload in patients with sickle cell disease. Haematologica. 2009;94:511-2. PubMed PMID: 70013481.

44. Mahfoud Z, Kassak K, Kreidieh K, Shamra S, Ramia S. Distribution of hepatitis C virus genotypes among injecting drug users in Lebanon. Virology journal. 2010;7:96. Epub 2010/05/15. doi: 10.1186/1743-422x-7-96. PubMed PMID: 20465784; PubMed Central PMCID: PMC2885342.

45. Naman RE, Mansour I, Klayme S, Khalil G. [Hepatitis C virus in hemodialysis patients and blood donors in Lebanon]. Le Journal medical libanais The Lebanese medical journal. 1996;44(1):4-9. Epub 1996/01/01. PubMed PMID: 8965318.

46. Ramia S, Koussa S, Taher A, Haraki S, Klayme S, Sarkis D, et al. Hepatitis-C-virus genotypes and hepatitis-G-virus infection in Lebanese thalassaemics. Annals of tropical medicine and parasitology. 2002;96(2):197-202. Epub 2002/06/26. doi: 10.1179/000349802125000439. PubMed PMID: 12080981.

47. Ramia S, Klayme S, Naman R. Infection with hepatitis B and C viruses and human retroviruses (HTLV-I and HIV) among high-risk Lebanese patients. Annals of tropical medicine and parasitology. 2003;97(2):187-92. Epub 2003/06/14. doi: 10.1179/000349803235001363. PubMed PMID: 12803874.

48. Dumaidi K, Al-Jawabreh A. Prevalence of occult HBV among hemodialysis patients in two districts in the northern part of the West Bank, Palestine. Journal of medical virology. 2014;86(10):1694-9. doi: 10.1002/jmv.24008. PubMed PMID: 24992542.

49. El-kader YE-OA, Elmanama AA, Ayesh BM. Prevalence and risk factors of hepatitis B and C viruses among haemodialysis patients in Gaza strip, Palestine. Virology journal. 2010;7:210. Epub 2010/09/03. doi: 10.1186/1743-422x-7-210. PubMed PMID: 20809985; PubMed Central PMCID: PMC2942824.

50. Stulhofer A, Chetty A, Rabie RA, Jwehan I, Ramlawi A. The prevalence of HIV, HBV, HCV, and HIV-related risk-taking behaviors among Palestinian injecting drug users in the East Jerusalem Governorate. Journal of urban health : bulletin of the New York Academy of Medicine. 2012;89(4):671-6. Epub 2012/06/08. doi: 10.1007/s11524-012-9672-z. PubMed PMID: 22674463; PubMed Central PMCID: PMC3535135.

51. Abdulkarim AS, Zein NN, Germer JJ, Kolbert CP, Kabbani L, Krajnik KL, et al. Hepatitis C virus genotypes and hepatitis G virus in hemodialysis patients from Syria: identification of two novel hepatitis C virus subtypes. The American journal of tropical medicine and hygiene. 1998;59(4):571-6. Epub 1998/10/28. PubMed PMID: 9790432.

52. Ali T, Schved JF. Registry of hemophilia and other bleeding disorders in Syria. Haemophilia. 2012;18(6):851-4. Epub 2012/06/08. doi: 10.1111/j.1365-2516.2012.02862.x. PubMed PMID: 22672010.

53. Syria Mental Health Directorate and National AIDS Programme (of Syrian Ministry of Health). Assessment of HIV risk and sero-prevalence among drug users in greater Damascus. Syrian Ministry of Health, United Nations Office on Drugs and Crime, and the Joint United Nations Programme on HIV/AIDS, 2008.

54. Moukeh G, Yacoub R, Fahdi F, Rastam S, Albitar S. Epidemiology of hemodialysis patients in Aleppo city. Saudi journal of kidney diseases and transplantation : an official publication of the Saudi Center for Organ Transplantation, Saudi Arabia. 2009;20(1):140-6. Epub 2008/12/30. PubMed PMID: 19112237.

55. Othman BM, Monem FS. Prevalence of hepatitis C virus antibodies among health care workers in Damascus, Syria. Saudi medical journal. 2001;22(7):603-5. Epub 2001/08/02. PubMed PMID: 11479642.
