# Supplementary material for: The Epidemiology of Hepatitis C Virus in the Fertile Crescent: Systematic Review and Meta-Analysis
Source: PLoS One. 2015 Aug 21;10(8):e0135281. doi: 10.1371/journal.pone.0135281 (PMC4546629; doi:10.1371/journal.pone.0135281)
Supplement: S7 Table — (DOCX) [file pone.0135281.s016.docx]

**S7 Table.** **Precision and risk of bias (ROB) assessment for individual hepatitis C virus (HCV) prevalence measures among the general population in countries of the Fertile Crescent.**

| **First author, year of publication [citation]** | **Years of data collection** | **Population** | **Sample size** | **HCV prev** | **Precis-ion** | **Study sampling procedure** | **HCV ascertain-ment** | **Response rate** |
| --- | --- | --- | --- | --- | --- | --- | --- | --- |
| **Iraq (n=45)** |  |  |  |  |  |  |  |  |
| Abdul-Aziz, 01 [[1](#_ENREF_1)] | 1999-01 | Blood donors | 20108 | 0.8% | High | High ROB | Low ROB | Low ROB |
| Abdul-Aziz, 01 [[1](#_ENREF_1)] | 1999-01 | New employees | 257 | 0.4% | High | High ROB | Low ROB | Low ROB |
| Abdullah, 12 [[2](#_ENREF_2)] | 2005-07 | Blood donors | 100 | 1% | High | High ROB | Low ROB | Low ROB |
| Al-Ani, 11 [[3](#_ENREF_3)] | 2007-09 | Children | 60 | 0% | Low | High ROB | Low ROB | Unclear^*^ |
| Al-Azzawi, 06 [[4](#_ENREF_4)] | 2004 | Pregnant women | 100 | 1% | High | High ROB | Low ROB | Unclear^*^ |
| Al-Doori, 06 [[5](#_ENREF_5)] | 2004-05 | Blood donors | 1978 | 0.4% | High | High ROB | Low ROB | Low ROB |
| Al-Duliami, 12 [[6](#_ENREF_6)] | 2010-11 | Blood donors | 90 | 0% | Low | Low ROB | Low ROB | Low ROB |
| Al-Greti, 13 [[7](#_ENREF_7)] | 2011-12 | General population | 50 | 4% | Low | High ROB | Low ROB | Unclear^*^ |
| Al-Hamdani, 12 [[8](#_ENREF_8)] | 2003-04, 06, 08-09 | New employees | 4162 | 2.6% | High | High ROB | Low ROB | Low ROB |
| Ali, 09 [[9](#_ENREF_9)] | 2007-08 | Blood donors | 600 | 1.7% | High | High ROB | Low ROB | Low ROB |
| Al-Jebori, 10 [[10](#_ENREF_10)] | 2003-09 | Mixed general population | 120460 | 0.2% | High | High ROB | Low ROB | Low ROB |
| Aljooani, 12 [[11](#_ENREF_11)] | 2005-07 | Blood donors | 430 | 2.8% | High | High ROB | Low ROB | Low ROB |
| Al-Juboury, 10 [[12](#_ENREF_12)] | 2007-08 | Blood donors | 23336 | 0.5% | High | High ROB | Low ROB | Low ROB |
| Al-Kamil, 11 [[13](#_ENREF_13)] | 2006-08 | Blood donors | 161987 | 0.1% | High | High ROB | Low ROB | Low ROB |
| Al-Kubaisy, 02 [[14](#_ENREF_14)] |  | Pregnant women | 3491 | 3.2% | High | Low ROB | Low ROB | Unclear^*^ |
| Al-Saad, 09 [[15](#_ENREF_15)] | 2008 | General population | 100 | 0% | High | High ROB | Low ROB | Unclear^*^ |
| Al Wtaify, 00 [[16](#_ENREF_16)] | 1998-99 | Outpatient hospital attendees (children) | 200 | 0.5% | High | Low ROB | Low ROB | Unclear^*^ |
| Al-Zamili, 09 [[17](#_ENREF_17)] | 2007-08 | Children | 325 | 0% | High | High ROB | Low ROB | Unclear^*^ |
| Amin, 12 [[18](#_ENREF_18)] | 2008-09 | Blood donors | 35540 | 0.1% | High | High ROB | Low ROB | Low ROB |
| Ataallah, 11 [[19](#_ENREF_19)] | 2006-09 | Blood donors | 495648 | 0.3% | High | High ROB | Low ROB | Low ROB |
| Chiad, 09 [[20](#_ENREF_20)] | 2003-04 | Blood donors | 1000 | 1% | High | High ROB | Low ROB | Low ROB |
| Chironna, 03 [[21](#_ENREF_21)] |  | Refugees | 637 | 0.1% | High | High ROB | Low ROB | Low ROB |
| Fadhil, 12 [[22](#_ENREF_22)] | 2010-11 | Outpatient hospital attendees | 50 | 0% | Low | High ROB | Low ROB | Unclear^*^ |
| Fawzi, 11 [[23](#_ENREF_23)] | 2010 | Pregnant women | 520 | 1.1% | High | High ROB | Low ROB | Unclear^*^ |
| Hamim, 12 [[24](#_ENREF_24)] | 2006-10 | Outpatient hospital attendees | 4886 | 0.9% | High | High ROB | Low ROB | Low ROB |
| Hamim, 12 [[25](#_ENREF_25)] | 2008-09 | Blood donors | 871973 | 0.4% | High | High ROB | Low ROB | Low ROB |
| Hassan, 08 [[26](#_ENREF_26)] | 1996-01 | Blood donors | 42140 | 0.1% | High | High ROB | Low ROB | Low ROB |
| Hussain, 08 [[27](#_ENREF_27)] | 2004-05 | General population | 108 | 0% | High | High ROB | Low ROB | Unclear^*^ |
| Hussein, 10 [[28](#_ENREF_28)] | 2006-07 | Blood donors | 31574 | 0.1% | High | High ROB | Low ROB | Low ROB |
| Hussain, 10 [[29](#_ENREF_29)] | 2008-09 | Blood donors | 117 | 0.8% | High | High ROB | Low ROB | Low ROB |
| Jassim, 11 [[30](#_ENREF_30)] |  | Blood donors | 170 | 0% | High | High ROB | Low ROB | Low ROB |
| Khalid, 12 [[31](#_ENREF_31)] |  | Blood donors | 100 | 2% | High | High ROB | Low ROB | Low ROB |
| Naji, 13 [[32](#_ENREF_32)] | 2011-12 | Newly married individuals | 200 | 1.5% | High | High ROB | Low ROB | Low ROB |
| Noaman, 12 [[33](#_ENREF_33)] | 2009-10 | Blood donors | 93 | 1.1% | Low | High ROB | Low ROB | Low ROB |
| Obied, 14 [[34](#_ENREF_34)] | 2012-13 | Blood donors | 5179 | 0.4% | High | High ROB | Low ROB | Low ROB |
| Omer, 11 [[35](#_ENREF_35)] | 2006-08 | General population | 98 | 7.1% | High | High ROB | Low ROB | Low ROB |
| Richter, 14 [[36](#_ENREF_36)] | 2011 | First generation immigrants | 290 | 0.3% | High | High ROB | Low ROB | High ROB |
| Saadoon, 08 [[37](#_ENREF_37)] | 2007 | Pregnant women | 875 | 5.1% | High | High ROB | Low ROB | Unclear^*^ |
| Saadoon, 12 [[38](#_ENREF_38)] |  | Blood donors | 1128 | 0.6% | High | High ROB | Low ROB | Low ROB |
| Salih, 07 [[39](#_ENREF_39)] | 2007 | Blood donors | 95 | 2.1% | Low | High ROB | Low ROB | Low ROB |
| Salman, 07 [[40](#_ENREF_40)] | 2006-07 | Pregnant women | 60 | 0% | Low | High ROB | Low ROB | Unclear^*^ |
| Tarky, 13 [[41](#_ENREF_41)] | 2005-06 | General population | 9610 | 0.4% | High | Low ROB | Low ROB | Low ROB |
| Tawfeeq, 13 [[42](#_ENREF_42)] | 2011-12 | Blood donors | 15560 | 0.3% | High | High ROB | Low ROB | Low ROB |
| Toffik, 06 [[43](#_ENREF_43)] |  | Outpatient clinic attendees | 100 | 0% | High | High ROB | Low ROB | Unclear^*^ |
| Toffik, 06 [[43](#_ENREF_43)] |  | Blood donors | 200 | 0.5% | High | High ROB | Low ROB | Unclear^*^ |
| **Jordan (n=5)** |  |  |  |  |  |  |  |  |
| Al Abbadi, 14 [[44](#_ENREF_44)] | 2009-11 | Blood donors | 94270 | 0.1% | High | High ROB | Low ROB | Low ROB |
| Al-Gani, 11 [[45](#_ENREF_45)] | 2006-09 | Blood donors | 8190 | 0.9% | High | High ROB | Low ROB | Low ROB |
| Hamoudi, 13 [[46](#_ENREF_46)] |  | Health centers’ attendees | 706 | 0.4% | High | Low ROB | Low ROB | Unclear^*^ |
| Jbara, 06 [[47](#_ENREF_47)] | 2003-05 | Blood donors | 8750 | 0.8% | High | High ROB | Low ROB | Low ROB |
| Rashdan, 08 [[48](#_ENREF_48)] | 2004-06 | Blood donors | 14236 | 0.2% | High | High ROB | Low ROB | Low ROB |
| **Lebanon (n=13)** |  |  |  |  |  |  |  |  |
| Araj, 95 [[49](#_ENREF_49)] |  | Blood donors | 4179 | 0.1% | High | Low ROB | Low ROB | Low ROB |
| Baddoura, 02 [[50](#_ENREF_50)] |  | General population (Lebanese) | 2879 | 0.6% | High | High ROB | Low ROB | Low ROB |
| Baddoura, 02 [[50](#_ENREF_50)] |  | General population (immigrants) | 103 | 2.9% | High | High ROB | Low ROB | Low ROB |
| Irani-Hakime, 01 [[51](#_ENREF_51)] | 1999 | Blood donors | 600 | 0.2% | High | High ROB | Low ROB | Low ROB |
| Irani-Hakime, 06 [[52](#_ENREF_52)] | 1997-03 | Blood donors | 16084 | 0.4% | High | High ROB | Low ROB | Low ROB |
| Nabulsi, 97 [[53](#_ENREF_53)] | 1993-95 | Pregnant women | 558 | 0% | High | High ROB | Low ROB | Unclear^*^ |
| Naman, 96 [[54](#_ENREF_54)] |  | Blood donors | 7771 | 0.4% | High | High ROB | Low ROB | Low ROB |
| Ramia, 03 [[55](#_ENREF_55)] |  | Blood donors | 500 | 0.2% | High | Low ROB | Low ROB | Low ROB |
| Ramia, 05 [[56](#_ENREF_56)] | 2002-03 | Blood donors | 56 | 0% | Low | Low ROB | Low ROB | Low ROB |
| Salem, 03 [[57](#_ENREF_57)] |  | Outpatient hospital attendees | 70 | 0% | Low | Low ROB | Low ROB | Unclear^*^ |
| Salem, 03 [[57](#_ENREF_57)] |  | Blood donors | 150 | 0% | High | Low ROB | Low ROB | Low ROB |
| Tamim, 01 [[58](#_ENREF_58)] | 1998-00 | Blood donors (Lebanese) | 5027 | 0.3% | High | High ROB | Low ROB | Low ROB |
| Tamim, 01 [[58](#_ENREF_58)] | 1998-00 | Blood donors (non-Lebanese) | 88 | 3.4% | Low | High ROB | Low ROB | Low ROB |
| **Palestine (n=11)** |  |  |  |  |  |  |  |  |
| PHIC, 14 [[59](#_ENREF_59)] | 2013 | Blood donors | 19571 | 0.2% | High | Unknown^**^ | Unknown^**^ | Unknown^**^ |
| PHIC, 14 [[59](#_ENREF_59)] | 2013 | Blood donors | 9643 | 0.3% | High | Unknown^**^ | Unknown^**^ | Unknown^**^ |
| MENA HIV ESP, 11  [[60](#_ENREF_60), [61](#_ENREF_61)] | 2011 | Blood donors | 63477 | 0.2% | High | Unknown^**^ | Unknown^**^ | Unknown^**^ |
| PHIC, 10 [[62](#_ENREF_62)] | 2009 | Blood donors | 48001 | 0.2% | High | Unknown^**^ | Unknown^**^ | Unknown^**^ |
| PHIC, 08 [[63](#_ENREF_63)] | 2007 | Blood donors | 49105 | 0.3% | High | Unknown^**^ | Unknown^**^ | Unknown^**^ |
| PHIC, 07 [[64](#_ENREF_64)] | 2006 | Blood donors | 53151 | 0.2% | High | Unknown^**^ | Unknown^**^ | Unknown^**^ |
| PHIC, 06 [[65](#_ENREF_65)] | 2005 | Blood donors | 45029 | 0.2% | High | Unknown^**^ | Unknown^**^ | Unknown^**^ |
| PHIC, 04 [[66](#_ENREF_66)] | 2003 | Blood donors | 44990 | 0.3% | High | Unknown^**^ | Unknown^**^ | Unknown^**^ |
| Novack, 07 [[67](#_ENREF_67)] | 1999-02 | Blood donors | 2784 | 3.9% | High | High ROB | Low ROB | Low ROB |
| Shemer-Avni, 98 [[68](#_ENREF_68)] |  | Blood donors | 1509 | 2.2% | High | High ROB | Low ROB | Low ROB |
| Shemer-Avni, 98 [[68](#_ENREF_68)] |  | Outpatient hospital attendees | 124 | 9% | High | Low ROB | Low ROB | Unclear^*^ |
| **Syria (n=10)** |  |  |  |  |  |  |  |  |
| Ali, 10 [[69](#_ENREF_69)] | 2000-07 | Blood donors | 131403 | 0.4% | High | High ROB | Low ROB | Low ROB |
| MENA HIV ESP, 10 [[60](#_ENREF_60), [61](#_ENREF_61)] | Q 1, 2006 | Blood donors | 55549 | 0.3% | High | Unknown^**^ | Unknown^**^ | Unknown^**^ |
| MENA HIV ESP, 10 [[60](#_ENREF_60), [61](#_ENREF_61)] | Q 2, 2006 | Blood donors | 87920 | 0.4% | High | Unknown^**^ | Unknown^**^ | Unknown^**^ |
| MENA HIV ESP, 10 [[60](#_ENREF_60), [61](#_ENREF_61)] | Q 3, 2006 | Blood donors | 77734 | 0.6% | High | Unknown^**^ | Unknown^**^ | Unknown^**^ |
| MENA HIV ESP, 10 [[60](#_ENREF_60), [61](#_ENREF_61)] | Q 4, 2006 | Blood donors | 83347 | 0.5% | High | Unknown^**^ | Unknown^**^ | Unknown^**^ |
| MENA HIV ESP, 10 [[60](#_ENREF_60), [61](#_ENREF_61)] | Q 1, 2007 | Blood donors | 83501 | 0.4% | High | Unknown^**^ | Unknown^**^ | Unknown^**^ |
| MENA HIV ESP, 10 [[60](#_ENREF_60), [61](#_ENREF_61)] | Q 2, 2007 | Blood donors | 92452 | 0.4% | High | Unknown^**^ | Unknown^**^ | Unknown^**^ |
| MENA HIV ESP, 10 [[60](#_ENREF_60), [61](#_ENREF_61)] | Q 3, 2007 | Blood donors | 83560 | 0.5% | High | Unknown^**^ | Unknown^**^ | Unknown^**^ |
| MENA HIV ESP, 10 [[60](#_ENREF_60), [61](#_ENREF_61)] | 2011 | Blood donors | 416984 | 0.6% | High | Unknown^**^ | Unknown^**^ | Unknown^**^ |
| Othman, 02 [[70](#_ENREF_70)] |  | Blood donors | 2100 | 0.9% | High | High ROB | Low ROB | Low ROB |

MENA HIV ESP, Middle East and North Africa HIV/AIDS Epidemiology Synthesis Project database; PHIC, Palestinian Health Information Center; Prev, prevalence; Q, quarter.

^*^Studies with missing information for any of the domains were classified as having unclear ROB for that specific domain.

^**^Studies extracted from regional databases with limited description of the sample not permitting the conduct of ROB assessment were classified as being of unknown quality.

**References**

1. Abdul-Aziz M, Abdul-Karem K, Shamse-El-Den S, Al-Moula GA. Prevalence of hepatitis B & C among people attending Kirkuk Public Health Laboratory. Al-Taqani. 2001;23(3):6-15.

2. Abdullah B. A., Khaled M. D., Maarouf M. N. Detection of hepatitis C virus (HCV) by ELISA, RIBA and Reverse Transcriptase- Polymerase Chain Reaction (RT-PCR) technique among kidney dialysis patients in Nineveh governorate/Iraq. Science Journal of Thi-Qar 2012;3(2):55-67.

3. Al-Ani M. H., Rasul T. H. Hepatitis B and C viral infections in children with acute leukemia in Erbil city. Journal of the Arab Board of Health Specializations. 2011;12(1):21-9.

4. Al-Azzawi M. K. K. Study of the spread of viral hepatitis pattern (C) among pregnant women in the city of Baquba. Journal of Diyala for Humanity Research 2006;(22):106-12.

5. Al-Doori A.M. Prevalence of hepatitis B and hepatitis C among blood donors in Al-Anbar governorate. Journal of the Faculty of Medicine of Baghdad. 2006;48(2):149-51.

6. Al-Duliami A. A., Al-Kiali K. K., Hasan AR. Is there any relationship between hepatitis C virus infection and skin diseases? Diyala Journal of Medicine. 2012;3(1):51-6.

7. Al-Greti S. H. H. Prevalence of hepatitis C virus in beta-thalassemia major patients at Karbala governorate. Journal of Babylon University for Pure and Applied Sciences. 2013;21(8):2801-5.

8. Al-Hamdani A.H., Al-Rawy S. K., Khamees H. A. Retrospective seroprevalence study of hepatitis B and C in Iraqi population at Baghdad: a hospital based study. Iraqi Journal of Community Medicine. 2012;(3):186-90.

9. Ali MK. Prevalence of hepatitis B and hepatitis C among blood donors in Baghdad, August 2007-December 2008. Journal of the Faculty of Medicine of Baghdad University. 2009;51(4):419-22.

10. Al-Jebori A. S., Hassan A. S., Al-Duliami A. A. Seroprevalence of hepatitis B and hepatitis C virus infections in Diyala province during 2003-2008. Diyala Journal For Pure Science. 2010;6(1):292-302.

11. Aljooani O. AA., Al-Hayani N. N., Mohammed M. J. The infection with HBV and HCV and their relationship to ABO blood group among blood donors. Journal of the Faculty of Medicine of Baghdad. 2012;54(1):52-6.

12. Al-Juboury A. W. F., Salih H. A. L. M., Al-Assadi M. K., Ali A. M. Seroprevalence of Hepatitis B and C among blood donors in Babylon Governorate-Iraq. Medical Journal of Babylon. 2010;7(1-2).

13. Al-Kamil E. A., Al-Yassin A. K. Transfusion transmitted diseases among blood donors of Basrah blood bank. Journal of the Arab Board of Health Specializations. 2011;12(1):35-40.

14. Al-Kubaisy WA, Niazi AD, Kubba K. History of miscarriage as a risk factor for hepatitis C virus infection in pregnant Iraqi women. Eastern Mediterranean health journal = La revue de sante de la Mediterranee orientale = al-Majallah al-sihhiyah li-sharq al-mutawassit. 2002;8(2-3):239-44. Epub 2004/09/02. PubMed PMID: 15339110.

15. Al-Saad T, Al-Alousi BM, Khalaf NM, Abdul Razak W. Prevalence of hepatitis B and C viruses among medical staff in Ramadi General Hospital. Al-Anbar Medical Journal. 2009;7(1):68-75.

16. Al Wtaify AS, Hassan MK. Prevalence of hepatitis C virus infection among multitransfused thalassemic children in Basrah - Iraq. Qatar Medical Journal. 2000;9(2):58-61. PubMed PMID: 2001134222.

17. Al-Zamili A. H., Al-Shebani A. M., Mohsin R. K. Prevalence of hepatitis C viral infection among multi-transfused thalassemic patients. Al-Qadisiah Medical Journal. 2009;5(7):89-99.

18. Amin RM. Prevalence of HBV and HCV in blood donors in Mosul city. Al-Taqani. 2012;25(2):70-7.

19. Ataallah TM, Hanan KA, Maysoun KS, Sadoon AA. Prevalence of hepatitis B and C among blood donors attending the National Blood Transfusion Center in Baghdad, Iraq from 2006-2009. Saudi medical journal. 2011;32(10):1046-50. Epub 2011/10/20. PubMed PMID: 22008925.

20. Chiad I. A., Al-Rubaii A., Al-Ani W., Al-Jaf A. A., Kadhem M. Sero-prevalence of hepatitis B virus, hepatitis C virus and human immuno deficiency virus infections among blood donors in the National Center of Blood Transfusion in Baghdad. Iraqi Journal of Community Medicine. 2009;22(4):215-7.

21. Chironna M, Germinario C, Lopalco PL, Carrozzini F, Barbuti S, Quarto M. Prevalence rates of viral hepatitis infections in refugee Kurds from Iraq and Turkey. Infection. 2003;31(2):70-4. Epub 2003/04/12. doi: 10.1007/s15010-002-3100-3. PubMed PMID: 12682810.

22. Fadhil R. S., Al-Khalidi S. J., Hussien S. M., Hasoon H. A., Hussien R. A., Alwan L. H. Complications of HBV, HCV and HIV infections in patients with Coolyze Anemia in Baghdad. Iraqi Journal of Cancer and Medical Genetics. 2012;5(1):59-63.

23. Fawzi N., Muhsin K. A. A. Screening for hepatitis B and hepatitis C viral infection among a sample of pregnant women in Al-Fallujah. Iraqi Medical Journal. 2011;57(2):119-25.

24. Hamim S. S. A survey study for the prevalence of hepatitis A, B and C in Thi-Qar province for 2006-2010. Journal of College of Education for Pure Science. 2012;2(1):262-77.

25. Hamim S. S. Seroprevalence of hepatitis B and C among blood voluntaries in Iraq for 2008 and 2009. Journal of College of Education for Pure Science. 2012;2(2):125-4.

26. Hassan A. S. Prevalence of anti-hepatitis C virus antibodies among blood donors and risky groups in Diyala. Journal of the Faculty of Medicine of Baghdad. 2008;50(4):467-70.

27. Hussain A. K., Mohammed S. H., Abd K. H. Screening of hepatitis B and C viral markers in Iraqi patients with proteinuria. Iraqi Postgraduate Medical Journal. 2008;7(4):362-8.

28. Hussein A. H. Seroprevalence of hepatitis B and C viruses among blood donors in Sulaimani Major Blood Bank for the years 2006 and 2007: a comparative study. Journal of Zankoy Sulaimani. 2010;13(1):15-20.

29. Hussain S. M., Hasan A. Does hepatitis C virus infection is a relevant factor for thyroid dysfunction. Diyala Journal for Pure Science. 2010;6(3):140-5.

30. Jassim A. N., Abdul-Hadi A. M., Al-Waysi S. A. Epidemiological study of viral hepatitis / Chibayish Marsh Reality in iraq. Journal of Baghdad for Science. 2011;8(1):23-8.

31. Khalid MD, Abdullah BA. Prevalence of anti-HCV antibodies among thalassemia patients in Mosul City, Iraq. Journal of Life Sciences. 2012;6(5):489-91.

32. Naji MY, Abdulzahra AA, Hmood AR. Prevalence of viral hepatitis B and C in newly married persons. Kufa Journal for Nursing Sciences. 2013;3(3).

33. Noaman N.G. Prevalence of hepatitis C virus infection among blood donors and certain risky groups in Diyala Province. Diyala Journal of Medicine. 2012;2(1):46-52.

34. Obied H.M., Alrodhan M.A., Mallah M.O. Molecular and immunological detection of hepatitis C virus infection among blood donors in Al-Muthanna province-Iraq. International Journal of Advanced Research. 2014;2(6):295-315.

35. Omer AR, Salih JI, Al-Nakshabandi AA. Frequency of blood-borne viral infections among leukemic patients in central Iraq. Saudi medical journal. 2011;32(1):55-61. Epub 2011/01/08. PubMed PMID: 21212918.

36. Richter C, Ter Beest G, Gisolf EH, P VANB, Waegemaekers C, Swanink C, et al. Screening for chronic hepatitis B and C in migrants from Afghanistan, Iran, Iraq, the former Soviet Republics, and Vietnam in the Arnhem region, The Netherlands. Epidemiology and infection. 2014:1-7. Epub 2014/01/09. doi: 10.1017/s0950268813003415. PubMed PMID: 24398373.

37. Saadoon I. H., Salih N. I., Saihood S. T. Hepatitis C virus infection and abortion among pregnant women in Mosul city. Medical Journal of Tikrit. 2008;1(141):161-4.

38. Saadoon A. A. Prevalence of viral hepatitis B and C among selected group in Thi-Qar. Thi-Qar Medical Journal. 2012;6(1):79-89.

39. Salih N. I., Saadoon I. H. Seroprevalence of hepatitis C virus among risk groups in Mosul city. Tikrit Journal of Pharmaceutical Sciences. 2007;3(2):138-41.

40. Salman Y. G. Serological cross reaction among some causative agents of women abortions (Toxoplasma gondii & Cytomegalo virus & Rubella virus), with the incidence of hepatitis virus (B & C). Tikret Journal of Pharmaceutical Sciences. 2007;3(2):102-11.

41. Tarky A. M., Akram W., Al-Naaimi A. S., Omer A. R. Epidemiology of viral heaptitis B and C in iraq: a national survey 2005-2006. Zanco Journal of Medical Sciences. 2013;17(1):370-80.

42. Tawfeeq WF. Detection of hepatitis C virus infection and genotypes among seropositive blood donors by polymerase chain reaction in babylon Governorate, Iraq. Medical Journal of Babylon. 2013;10(1):25-37.

43. Toffik K. A., Al-Diwan J. K., Al-Hadithi T. S., Al-Waiz M. M., Omer A. R. Prevalence of the serological markers of hepatitis B, C, and D among patients with sexually transmitted diseases (STDs) in Baghdad, Iraq. Journal of the Arab Board of Medical Specializations. 2006;8(1):105-9.

44. Al Abbadi B., Al Amr M., Abasi L., Saleem A., Abu Hazeem N., Marafi A. Seroprevalence of HBV, HCV, HIV and syphilis infections among blood donors at Blood Bank of King Hussein Medical Center: a 3 year study. Middle East Journal of Family Medicine 2014;12(6):10-3.

45. Al-Gani FA. Prevalence of HBV, HCV, and HIV-1, 2 infections among blood donors in Prince Rashed Ben Al-Hassan Hospital in North region of Jordan. International Journal of Biological & Medical Research. 2011;2(4):912-6.

46. Hamoudi W, Ali SA, Abdallat M, Estes CR, Razavi HA. HCV infection prevalence in a population recruited at health centers in Jordan. Journal of epidemiology and global health. 2013;3(2):67-71. Epub 2013/07/17. doi: 10.1016/j.jegh.2013.02.003. PubMed PMID: 23856567.

47. Jbara I, Abu Alshiekh NK, Almomani AM, Khasawneh RH, Omari AK. Prevalence of hepatitis C virus antibodies among blood donors at Prince Hashem Hospital, Zarka- Jordan. Jordan Medical Journal. 2006;40(3):190-3. PubMed PMID: 2007107899.

48. Rashdan A, Hijjawi S, Jadallah K, Matalka I. Prevalence of hepatitis C virus antibodies among blood donors in Northern Jordan. Jordan Medical Journal. 2008;42(3):179-83. PubMed PMID: 2009005380.

49. Araj GF, Kfoury-Baz EE, Barada KA, Nassif RE, Alami SY. Hepatitis C virus : prevalence in Lebanese blood donors and brief overview of the disease. Le Journal medical libanais The Lebanese medical journal. 1995;43(1):11-6. Epub 1995/01/01. PubMed PMID: 8676356.

50. Baddoura R, Haddad C, Germanos M. Hepatitis B and C seroprevalence in the Lebanese population. Eastern Mediterranean health journal = La revue de sante de la Mediterranee orientale = al-Majallah al-sihhiyah li-sharq al-mutawassit. 2002;8(1):150-6. Epub 2004/08/28. PubMed PMID: 15330570.

51. Irani-Hakime N, Aoun J, Khoury S, Samaha HR, Tamim H, Almawi WY. Seroprevalence of hepatitis C infection among health care personnel in Beirut, Lebanon. American journal of infection control. 2001;29(1):20-3. Epub 2001/02/15. PubMed PMID: 11172314.

52. Irani-Hakime N, Musharrafieh U, Samaha H, Almawi WY. Prevalence of antibodies against hepatitis B virus and hepatitis C virus among blood donors in Lebanon, 1997-2003. American journal of infection control. 2006;34(4):241-3. Epub 2006/05/09. doi: 10.1016/j.ajic.2005.06.009. PubMed PMID: 16679184.

53. Nabulsi MM, Araj GF, Farah AE, Khalil AM. Hepatitis C virus antibodies in pregnant Lebanese women. Journal of obstetrics and gynaecology : the journal of the Institute of Obstetrics and Gynaecology. 1997;17(6):548. Epub 1997/01/01. doi: 10.1080/01443619768560. PubMed PMID: 15511954.

54. Naman RE, Mansour I, Klayme S, Khalil G. [Hepatitis C virus in hemodialysis patients and blood donors in Lebanon]. Le Journal medical libanais The Lebanese medical journal. 1996;44(1):4-9. Epub 1996/01/01. PubMed PMID: 8965318.

55. Ramia S, Klayme S, Naman R. Infection with hepatitis B and C viruses and human retroviruses (HTLV-I and HIV) among high-risk Lebanese patients. Annals of tropical medicine and parasitology. 2003;97(2):187-92. Epub 2003/06/14. doi: 10.1179/000349803235001363. PubMed PMID: 12803874.

56. Ramia S, Ramlawi F, Kanaan M, Klayme S, Naman R. Frequency and significance of antibodies against hepatitis B core (anti-HBc) antigen as the only serological marker for hepatitis B infection in Lebanese blood donors. Epidemiology and infection. 2005;133(4):695-9. Epub 2005/07/30. PubMed PMID: 16050516; PubMed Central PMCID: PMC2870298.

57. Salem Z, Nuwaiyri-Salti N, Ramlawi F, Ramia S. Hepatitis C virus infection in Lebanese patients with B-cell non-Hodgkin's lymphoma. European journal of epidemiology. 2003;18(3):251-3. Epub 2003/06/13. doi: 10.1023/a:1023380316098. PubMed PMID: 12800950.

58. Tamim H, Irani-Hakime N, Aoun JP, Khoury S, Samaha H, Almawi WY. Seroprevalence of hepatitis C virus (HCV) infection among blood donors: a hospital-based study. Transfusion and apheresis science : official journal of the World Apheresis Association : official journal of the European Society for Haemapheresis. 2001;24(1):29-35. Epub 2001/08/23. PubMed PMID: 11515608.

59. Palestinian Health Information Center. Health Annual Report 2013. URL: <http://www.moh.ps/?lang=1&page=4&id=939:> 2014.

60. Abu-Raddad L, Akala FA, Semini I, Riedner G, Wilson D, Tawil O. Characterizing the HIV/AIDS epidemic in the Middle East and North Africa : Time for strategic action. World Bank/UNAIDS/WHO Publication, editor. Washington DC: The World Bank Press; 2010.

61. Additional country-level data provided through the MENA HIV/AIDS Epidemiology Synthesis Project database by the World Health Organization Regional Office for the Eastern Mediterranean. 2014.

62. Palestinian Health Information Center. Health Annual Report 2009. URL: <http://www.moh.ps/?lang=1&page=4&id=150:> 2010.

63. Palestinian Health Information Center. Health Annual Report 2007. URL: <http://www.moh.ps/?lang=1&page=4&id=139:> 2008.

64. Palestinian Health Information Center. Health Annual Report 2006. URL: <http://www.moh.ps/?lang=1&page=4&id=142:> 2007.

65. Palestinian Health Information Center. Health Annual Report 2005. URL: <http://www.moh.ps/?lang=1&page=4&id=9:> 2006.

66. Palestinian Health Information Center. Health Annual Report 2003. URL: <http://www.moh.ps/?lang=1&page=4&id=137:> 2004.

67. Novack L, Shinar E, Safi J, Soliman H, Yaari A, Galai N, et al. Evaluation of pooled screening for anti-HCV in two blood services set-ups. Tropical medicine & international health : TM & IH. 2007;12(3):415-21. Epub 2007/02/23. doi: 10.1111/j.1365-3156.2006.01810.x. PubMed PMID: 17313513.

68. Shemer-Avni Y, el Astal Z, Kemper O, el Najjar KJ, Yaari A, Hanuka N, et al. Hepatitis C virus infection and genotypes in Southern Israel and the Gaza Strip. Journal of medical virology. 1998;56(3):230-3. Epub 1998/10/23. PubMed PMID: 9783690.

69. Ali T, Daher N. Prevalence des infections transmissibles par transfusion chez les donneurs de sang au centre de transfusion sanguine de l'universite de Damas et l'importance des mesures de la securite transfusionelle. Damscus University Journal of Health Sciences. 2010;26(1):107-23.

70. Othman BM, Monem FS. Prevalence of hepatitis C virus antibodies among intravenous drug abusers and prostitutes in Damascus, Syria. Saudi medical journal. 2002;23(4):393-5. Epub 2002/04/16. PubMed PMID: 11953762.
