# Supplementary material for: The Epidemiology of Hepatitis C Virus in the Fertile Crescent: Systematic Review and Meta-Analysis
Source: PLoS One. 2015 Aug 21;10(8):e0135281. doi: 10.1371/journal.pone.0135281 (PMC4546629; doi:10.1371/journal.pone.0135281)
Supplement: S8 Table — (DOCX) [file pone.0135281.s017.docx]

**S8 Table.** **Precision and risk of bias (ROB) assessment of individual hepatitis C virus (HCV) prevalence measures among populations at intermediate risk and among special clinical populations in countries of the Fertile Crescent.**

| **First author, year of publication [citation]** | **Years of data collection** | **Population** | **Sample size** | **HCV prev** | **Precis-ion** | **Study sampling procedure** | **HCV ascertain-ment** | **Response rate** |
| --- | --- | --- | --- | --- | --- | --- | --- | --- |
| **Iraq** |  |  |  |  |  |  |  |  |
| ***Intermediate risk (n=18)*** |  |  |  |  |  |  |  |  |
| Abdul-Aziz, 01 [[1](#_ENREF_1)] | 1999-01 | Household contacts of index patients | 433 | 1.4% | High | High ROB | Low ROB | Low ROB |
| Abdul-Aziz, 01 [[1](#_ENREF_1)] | 1999-01 | Health care workers | 219 | 0% | High | High ROB | Low ROB | Low ROB |
| Abdul-Aziz, 01 [[1](#_ENREF_1)] | 1999-01 | Midwives | 196 | 0% | High | High ROB | Low ROB | Low ROB |
| Abdul-Aziz, 01 [[1](#_ENREF_1)] | 1999-01 | Barbers | 566 | 0.3% | High | High ROB | Low ROB | Low ROB |
| Albaitushi, 11 [[2](#_ENREF_2)] |  | Type 1 diabetes mellitus patients | 54 | 35.1% | Low | High ROB | Low ROB | Unclear^*^ |
| Al-Hawaz, 14 [[3](#_ENREF_3)] | 2012-13 | Hospitalized patients (pre-operative surgery) | 1730 | 2.5% | High | High ROB | Low ROB | Low ROB |
| Al-Marzoqi, 09 [[4](#_ENREF_4)] | 2008 | Hospitalized outpatient populations (presenting to emergency ward) | 50 | 14% | Low | High ROB | Low ROB | Unclear^*^ |
| Al-Mashhadani, 07 [[5](#_ENREF_5)] | 2002 | Health care workers | 60 | 1.6% | Low | High ROB | Low ROB | Unclear^*^ |
| Al-Mashhadani, 09 [[6](#_ENREF_6)] | 1995-98 | Health care workers | 1656 | 1.5% | High | High ROB | Low ROB | Unclear^*^ |
| Al-Saad, 09 [[7](#_ENREF_7)] | 2008 | Health care workers | 419 | 0% | High | High ROB | Low ROB | Unclear^*^ |
| Hassan, 08 [[8](#_ENREF_8)] | 1996-01 | Health care workers | 649 | 0.8% | High | High ROB | Low ROB | Low ROB |
| Hassan, 08 [[8](#_ENREF_8)] | 1996-01 | Midwives | 384 | 0.3% | High | High ROB | Low ROB | Low ROB |
| Hassan, 08 [[8](#_ENREF_8)] | 1996-01 | Prisoners | 625 | 0.6% | High | High ROB | Low ROB | Low ROB |
| Hassan, 08 [[8](#_ENREF_8)] | 1996-01 | Household contacts of index patients | 486 | 1.2% | High | High ROB | Low ROB | Low ROB |
| Hussain, 08 [[9](#_ENREF_9)] | 2003-07 | Dental providers | 450 | 9.1% | High | High ROB | High ROB | Unclear^*^ |
| Noaman, 12 [[10](#_ENREF_10)] | 2009-10 | Health care workers | 90 | 3.3% | Low | High ROB | Low ROB | High ROB |
| Saadoon, 12 [[11](#_ENREF_11)] |  | Health care workers | 100 | 0% | High | High ROB | Low ROB | Low ROB |
| Toffik, 06 [[12](#_ENREF_12)] |  | Sexually transmitted infections patients | 163 | 1.2% | High | High ROB | Low ROB | Unclear^*^ |
| ***Special clinical populations (n=26)*** |  |  |  |  |  |  |  |  |
| Abass, 08 [[13](#_ENREF_13)] | 2005-07 | Chronic liver disease patients | 1990 | 3.8% | High | High ROB | Low ROB | Low ROB |
| Abbas, 11 [[14](#_ENREF_14)] | 2010-11 | Hospitalized patients symptomatic for viral hepatitis | 964 | 0.2% | High | High ROB | Low ROB | Low ROB |
| Abbas, 11 [[14](#_ENREF_14)] | 2010-11 | Outpatients symptomatic for viral hepatitis | 455 | 1.1% | High | High ROB | Low ROB | Low ROB |
| Abdul-Aziz, 01 [[1](#_ENREF_1)] | 1999-01 | Acute viral hepatitis patients | 875 | 3.4% | High | High ROB | Low ROB | Low ROB |
| Al-Ali, 14 [[15](#_ENREF_15)] | 2006-07 | Cancer patients on chemotherapy (children) | 85 | 0% | Low | High ROB | Low ROB | Unclear^*^ |
| Al-Ani, 11 [[16](#_ENREF_16)] | 2007-09 | Leukemia patients (pre-chemotherapy- children) | 60 | 6.7% | Low | High ROB | Low ROB | Unclear^*^ |
| Al-Dulaimi, 12 [[17](#_ENREF_17)] | 2010-11 | Chronic renal failure patients (pre-dialysis) | 68 | 0% | Low | High ROB | Low ROB | Unclear^*^ |
| Al-Duliami, 12 [[18](#_ENREF_18)] | 2010-11 | Skin disease patients | 200 | 1% | High | High ROB | Low ROB | Unclear^*^ |
| Al-Hamdani, 12 [[19](#_ENREF_19)] | 2003-04, 06, 08-09 | Patients with liver complaints | 7560 | 3.8% | High | High ROB | Low ROB | Low ROB |
| Al-Jadiry, 08 [[20](#_ENREF_20)] | 2007 | Acute lymphoblastic leukemia patients | 148 | 0% | High | High ROB | Low ROB | Low ROB |
| Al-Janabi, 12 [[21](#_ENREF_21)] | 2010 | Acute viral hepatitis patients | 185 | 71.9% | High | High ROB | Low ROB | Unclear^*^ |
| Al-Kubaisy, 14 [[22](#_ENREF_22)] | 2000-03 | Hepatocellular carcinoma patients | 65 | 26.1% | Low | High ROB | Low ROB | Unclear^*^ |
| Al-Kubaisy, 14 [[22](#_ENREF_22)] | 2000-03 | Patients with malignant tumors | 82 | 11% | Low | High ROB | Low ROB | Unclear^*^ |
| Ali, 13 [[23](#_ENREF_23)] | 2011-12 | Rheumatoid arthritis patients | 60 | 1.7% | Low | High ROB | Low ROB | Unclear^*^ |
| Al-Obeidy, 10 [[24](#_ENREF_24)] | 2008-09 | Chronic liver disease patients | 55 | 36.4% | Low | High ROB | Low ROB | Unclear^*^ |
| Alshareefy, 13 [[25](#_ENREF_25)] | 2012-13 | Liver failure patients | 127 | 26.8% | High | High ROB | Low ROB | Low ROB |
| Hassan, 08 [[8](#_ENREF_8)] | 1996-01 | Acute icteric/jaundice patients | 1656 | 2.1% | High | High ROB | Low ROB | Low ROB |
| Hussain, 08 [[26](#_ENREF_26)] | 2004-05 | Proteinuria patients | 143 | 6.3% | High | High ROB | Low ROB | Unclear^*^ |
| Hussain, 10 [[27](#_ENREF_27)] | 2008-09 | Patients with thyroid disorders | 122 | 1.6% | High | High ROB | Low ROB | Unclear^*^ |
| Hussein, 12 [[28](#_ENREF_28)] | 2010-11 | Suspected cases of viral hepatitis | 214 | 3.7% | High | High ROB | Low ROB | Low ROB |
| Joda, 12 [[29](#_ENREF_29)] |  | Patients with chronic viral hepatitis | 50 | 62% | Low | High ROB | Low ROB | Unclear^*^ |
| Khaleel, 14 [[30](#_ENREF_30)] | 2010 | Suspected acute viral hepatitis cases | 2692 | 0.7% | High | Low ROB | Low ROB | Low ROB |
| Saadoon, 12 [[11](#_ENREF_11)] |  | Acute viral hepatitis | 126 | 4% | High | High ROB | Low ROB | Low ROB |
| Saeed, 09 [[31](#_ENREF_31)] | 2007-08 | Chronic liver disease patients | 50 | 6% | Low | High ROB | Low ROB | Unclear^*^ |
| Salman, 07 [[32](#_ENREF_32)] | 2006-07 | Pregnant women with high risk of delivery complications | 343 | 1.7% | High | High ROB | Low ROB | Unclear^*^ |
| Zgair, 07 [[33](#_ENREF_33)] |  | Acute viral hepatitis patients | 355 | 0% | High | High ROB | Low ROB | Low ROB |
| **Jordan** |  |  |  |  |  |  |  |  |
| ***Intermediate risk (n=1)*** |  |  |  |  |  |  |  |  |
| Al-Quadan, 02 [[34](#_ENREF_34)] | 1999 | Health care workers | 152 | 0.6% | High | High ROB | Low ROB | Unclear^*^ |
| ***Special clinical populations (n=1)*** |  |  |  |  |  |  |  |  |
| Al-Sheyyab, 01 [[35](#_ENREF_35)] | 1998-99 | Patients with hemolytic anemia | 143 | 40.5% | High | High ROB | Low ROB | Low ROB |
| ***Mixed populations (n=1)*** |  |  |  |  |  |  |  |  |
| Al-Quadan, 02 [[34](#_ENREF_34)] | 1999 | Mixed population | 426 | 4.5% | High | High ROB | Low ROB | Unclear^*^ |
| **Lebanon** |  |  |  |  |  |  |  |  |
| ***Intermediate risk (n=5)*** |  |  |  |  |  |  |  |  |
| Irani-Hakime, 01 [[36](#_ENREF_36)] | 1999 | Health care workers | 500 | 0.4% | High | High ROB | Low ROB | Low ROB |
| Kassak, 11 [[37](#_ENREF_37)] | 2007-08 | Men who have sex with men | 101 | 0% | High | Low ROB | Low ROB | Low ROB |
| Kassak, 11 [[37](#_ENREF_37)] | 2007-08 | Female sex workers | 103 | 0% | High | Low ROB | Low ROB | Low ROB |
| Mahfoud, 10 [[38](#_ENREF_38)] | 2007-08 | Prisoners | 350 | 3.4% | High | Low ROB | Low ROB | High ROB |
| Ramia, 04 [[39](#_ENREF_39)] |  | HIV infected patients (sexual route) | 90 | 7.7% | Low | High ROB | Low ROB | Unclear^*^ |
| ***Special clinical populations (n=3)*** |  |  |  |  |  |  |  |  |
| Otrock, 13 [[40](#_ENREF_40)] | 2007 | Patients with lymphomas (Hodgkin & non-Hodgkin) | 122 | 0% | High | High ROB | Low ROB | High ROB |
| Salem, 03 [[41](#_ENREF_41)] |  | Patients with malignancies (other than non-Hodgkin lymphoma) | 60 | 0% | Low | High ROB | Low ROB | Unclear^*^ |
| Yaghi, 06 [[42](#_ENREF_42)] | 1998-03 | Hepatocellular carcinoma patients | 92 | 19.6% | Low | High ROB | Low ROB | Low ROB |
| **Palestine** |  |  |  |  |  |  |  |  |
| ***Mixed populations (n=3)*** |  |  |  |  |  |  |  |  |
| MENA HIV ESP, 11 [[43](#_ENREF_43), [44](#_ENREF_44)] | 2011 | Patients and contacts | 20889 | 1.7% | High | Unknown^**^ | Unknown^**^ | Unknown^**^ |
| PHIC, 07 [[45](#_ENREF_45)] | 2006 | Patients and contacts | 16376 | 2.2% | High | Unknown^**^ | Unknown^**^ | Unknown^**^ |
| PHIC, 04 [[46](#_ENREF_46)] | 2003 | Patients and contacts | 12398 | 4.6% | High | Unknown^**^ | Unknown^**^ | Unknown^**^ |
| **Syria** |  |  |  |  |  |  |  |  |
| ***Intermediate risk (n=3)*** |  |  |  |  |  |  |  |  |
| Syrian MOH, date unknown [[47](#_ENREF_47)] |  | Health care workers | 400 | 3.8% | High | Low ROB | High ROB | Unclear^*^ |
| Othman, 01 [[48](#_ENREF_48)] |  | Health care workers | 189 | 3% | High | High ROB | Low ROB | Unclear^*^ |
| Othman, 02 [[49](#_ENREF_49)] |  | Female sex workers | 102 | 2% | High | High ROB | Low ROB | Unclear^*^ |
| ***Special clinical populations (n=1)*** |  |  |  |  |  |  |  |  |
| Al-Azmeh, 99 [[50](#_ENREF_50)] | 1995-98 | Acute viral hepatitis patients | 193 | 1.0% | High | High ROB | Low ROB | Unclear^*^ |

MENA HIV ESP, Middle East and North Africa HIV/AIDS Epidemiology Synthesis Project database; MOH, Ministry of Health; PHIC, Palestinian Health Information Center; Prev, prevalence.

^*^Studies with missing information for any of the domains were classified as having unclear ROB for that specific domain.

^**^Studies extracted from regional databases with limited description of the sample not permitting the conduct of ROB assessment were classified as being of unknown quality.

**References**

1. Abdul-Aziz M, Abdul-Karem K, Shamse-El-Den S, Al-Moula GA. Prevalence of hepatitis B & C among people attending Kirkuk Public Health Laboratory. Al-Taqani. 2001;23(3):6-15.

2. Albaitushi A., Tariq G., Mahmood M. Viral infections and diabetes mellitus. Journal of the Biotechnology Research Center. 2011;5(3):29-33.

3. Al-Hawaz M. H., Al-Hijaj M. H., Al-Mansori S. A. Prevalence of hepatitis B and hepatitis C among preoperative surgical patients at Basrah General Hospital. Basrah Journal of Surgery. 2014;20(1):62-5.

4. Al-Marzoqi A. H., Shemmran A. R., Al-Hindi Z., Al-Taee Z. M., Al-Nafee' M. K. Bacterial and viral infections associated with thalassemia in Hillah city. Journal of Al-Qadisiyah for Pure Science. 2009;14(3):1-15.

5. Al-Mashhadani JI. Hepatitis C virus infection among haemodialysis patients in Al-Anbar governorate. Iraqi Journal of Community Medicine. 2007;20(1):20-3.

6. Al-Mashhadani J. I., Al-Hadithi T. S., Al-Diwan J. K., Omer A. R. Sociodemographic characteristics and risk factors of hepatitis B and C among Iraqi health care workers. Journal of the Faculty of Medicine of Baghdad. 2009;51(3):308-11.

7. Al-Saad T, Al-Alousi BM, Khalaf NM, Abdul Razak W. Prevalence of hepatitis B and C viruses among medical staff in Ramadi General Hospital. Al-Anbar Medical Journal. 2009;7(1):68-75.

8. Hassan A. S. Prevalence of anti-hepatitis C virus antibodies among blood donors and risky groups in Diyala. Journal of the Faculty of Medicine of Baghdad. 2008;50(4):467-70.

9. Hussain AI, Anizy HH. Prevalence of viral hepatitis B and C among dentistry professionals in Anbar province. Journal of Al-Anbar University for Pure Science. 2008;2(2):18-24.

10. Noaman N.G. Prevalence of hepatitis C virus infection among blood donors and certain risky groups in Diyala Province. Diyala Journal of Medicine. 2012;2(1):46-52.

11. Saadoon A. A. Prevalence of viral hepatitis B and C among selected group in Thi-Qar. Thi-Qar Medical Journal. 2012;6(1):79-89.

12. Toffik K. A., Al-Diwan J. K., Al-Hadithi T. S., Al-Waiz M. M., Omer A. R. Prevalence of the serological markers of hepatitis B, C, and D among patients with sexually transmitted diseases (STDs) in Baghdad, Iraq. Journal of the Arab Board of Medical Specializations. 2006;8(1):105-9.

13. Abass Y. A., Al-Husseiny K. R., Kareem A. A. Epidemiology of hepatitis HBV and HCV at Thi-Qar province - Iraq. Al-Qadisiah Medical Journal. 2008;4(5):160-71.

14. Abbas F. N. Use ELISA technique to detect viral hepatitis in Thi-Qar province: a retrospective study. Thi-Qar Medical Journal. 2011;5(3):71-6.

15. Al-Ali N. AA., AL-Kayatt T. N. Seroprevalence of hepatitis B & C in pediatric malignancies. Iraqi Postgraduate Medical Journal. 2014;13(2):262-7.

16. Al-Ani M. H., Rasul T. H. Hepatitis B and C viral infections in children with acute leukemia in Erbil city. Journal of the Arab Board of Health Specializations. 2011;12(1):21-9.

17. Al-Dulaimi S. B. K., Al-Ubadi A. E., Al-Ubadi A. E., Al-Bayatti E. N., Al-Saday S. D. K. Toxoplasma gondii, HCV, and HBV seroprevalence in Haemodialysis patients with chronic renal failure in Al-Kindy Hospital Baghdad, Iraqi. Al-Mustansiriyah Journal of Science. 2012;23(5):33-8.

18. Al-Duliami A. A., Al-Kiali K. K., Hasan AR. Is there any relationship between hepatitis C virus infection and skin diseases? Diyala Journal of Medicine. 2012;3(1):51-6.

19. Al-Hamdani A.H., Al-Rawy S. K., Khamees H. A. Retrospective seroprevalence study of hepatitis B and C in Iraqi population at Baghdad: a hospital based study. Iraqi Journal of Community Medicine. 2012;(3):186-90.

20. Al-Jadiry M. Viral hepatitis markers screen in children with acute lymphoblastic leukemia experience of Children Welfare Teaching Hospital. Journal of the Faculty of Medicine of Baghdad. 2008;50(2):223-30.

21. Al-Janabi D. K. F., Hamzah N. A. Epidemiological, immunological and physiological study on viral hepatitis types B, C and E viruses in Babylon province. Journal of Al-Qadisiyah for Pure Science. 2012;17(4):1-9.

22. Al-Kubaisy W. A., Obaid K. J., Noor N. M., Ibrahim N. S., Al-Azawi A. A. Hepatitis C virus prevalence and genotyping among hepatocellular carcinoma patients in Baghdad. Asian pacific Journal of Cancer Prevention. 2014;15(18):7725-30.

23. Ali Q. R., Alani M. Q., Abdallah H. N. Evaluate the prevalence of CCP and RF antibodies as a marker for diagnosis and progression of Rheumatoid Arthritis disease and assess the prevalence of HCV in RA patients. . Journal of Al-Anbar University for Pure Science. 2013;7(2).

24. Al-Obeidy E., Abdullah S. F., Mukhlis F. A. Hepatitis G virus infection among Iraqi patients with chronic liver diseases. Journal of the Faculty of Medicine of Baghdad. 2010;52(3):299-303.

25. Alshareefy A. A. In Iraqi cirrhotic patients, can oesophageal variceal bleeding make child plugh score more predictive of outcome and mortality. Medical Journal of Babylon. 2013;10(3):590-9.

26. Hussain A. K., Mohammed S. H., Abd K. H. Screening of hepatitis B and C viral markers in Iraqi patients with proteinuria. Iraqi Postgraduate Medical Journal. 2008;7(4):362-8.

27. Hussain S. M., Hasan A. Does hepatitis C virus infection is a relevant factor for thyroid dysfunction. Diyala Journal for Pure Science. 2010;6(3):140-5.

28. Hussein N. J. Studying the prevalence of hepatitis virus in patients in Sammawa city. Journal of Kerbala University. 2012;10(4):116-24.

29. Joda A. T. Rheumatological manifestations of chronic viral hepatitis. Al-Mustansiriyah Medical Journal. 2012;11(2):1-6.

30. Khaleel H. A., Turky A. M., Al-Naaimi A. S., Jalil R. W., Mekhlef O. A., Abdul Kareem S., et al. Prevalence of HBsAg and anti HCV Ab among patients with suspected acute viral hepatitis in Baghdad, Iraq in 2010. Epidemiology Reports. 2014;1(1). doi: 10.7243/2054-9911-1-1

31. Saeed B. N., Hakeam S., Ahmed L., Ahmed J. The value of using echocardiography in patients of advanced liver disease with cardio pulmonary complications. The Iraqi Postgraduate Medical Journal. 2009;8(4):323-6.

32. Salman Y. G. Serological cross reaction among some causative agents of women abortions (Toxoplasma gondii & Cytomegalo virus & Rubella virus), with the incidence of hepatitis virus (B & C). Tikret Journal of Pharmaceutical Sciences. 2007;3(2):102-11.

33. Zgair A. K., Ali L. K., Zgair M. K., Eissa R. H. The frequency of IgM-anti HAV in the sera of patients with hepatitis in Iraq. Journal of Baghdad for Science. 2007;4(2):298-300.

34. Quadan A. Prevalence of anti hepatitis C virus among the hospital populations in Jordan. The new microbiologica. 2002;25(3):269-73. Epub 2002/08/14. PubMed PMID: 12173766.

35. Al-Sheyyab M, Batieha A, El-Khateeb M. The prevalence of hepatitis B, hepatitis C and human immune deficiency virus markers in multi-transfused patients. Journal of tropical pediatrics. 2001;47(4):239-42. Epub 2001/08/29. PubMed PMID: 11523766.

36. Irani-Hakime N, Aoun J, Khoury S, Samaha HR, Tamim H, Almawi WY. Seroprevalence of hepatitis C infection among health care personnel in Beirut, Lebanon. American journal of infection control. 2001;29(1):20-3. Epub 2001/02/15. PubMed PMID: 11172314.

37. Kassak K, Mahfoud Z, Kreidieh K, Shamra S, Afifi R, Ramia S. Hepatitis B virus and hepatitis C virus infections among female sex workers and men who have sex with men in Lebanon: prevalence, risk behaviour and immune status. Sexual health. 2011;8(2):229-33. Epub 2011/05/20. doi: 10.1071/sh10080. PubMed PMID: 21592438.

38. Mahfoud Z, Kassak K, Kreidieh K, Shamra S, Ramia S. Prevalence of antibodies to human immunodeficiency virus (HIV), hepatitis B and hepatitis C and risk factors in prisoners in Lebanon. Journal of infection in developing countries. 2010;4(3):144-9. Epub 2010/03/31. PubMed PMID: 20351454.

39. Ramia S, Mokhbat J, Sibai A, Klayme S, Naman R. Exposure rates to hepatitis C and G virus infections among HIV-infected patients: evidence of efficient transmission of HGV by the sexual route. International journal of STD & AIDS. 2004;15(7):463-6. Epub 2004/07/02. doi: 10.1258/0956462041211180. PubMed PMID: 15228731.

40. Otrock ZK, Saab J, Aftimos G, Nasr F, Farhat FS, Khairallah S, et al. A collaborative nationwide lymphoma study in Lebanon: incidence of various subtypes and analysis of associations with viruses. Pathology oncology research : POR. 2013;19(4):715-22. Epub 2013/05/09. doi: 10.1007/s12253-013-9634-0. PubMed PMID: 23653112.

41. Salem Z, Nuwaiyri-Salti N, Ramlawi F, Ramia S. Hepatitis C virus infection in Lebanese patients with B-cell non-Hodgkin's lymphoma. European journal of epidemiology. 2003;18(3):251-3. Epub 2003/06/13. doi: 10.1023/a:1023380316098. PubMed PMID: 12800950.

42. Yaghi C, Sharara AI, Rassam P, Moucari R, Honein K, BouJaoude J, et al. Hepatocellular carcinoma in Lebanon: Etiology and prognostic factors associated with short-term survival. World Journal of Gastroenterology. 2006;12(22):3575-80. PubMed PMID: 2006318933.

43. Abu-Raddad L, Akala FA, Semini I, Riedner G, Wilson D, Tawil O. Characterizing the HIV/AIDS epidemic in the Middle East and North Africa : Time for strategic action. World Bank/UNAIDS/WHO Publication, editor. Washington DC: The World Bank Press; 2010.

44. Additional country-level data provided through the MENA HIV/AIDS Epidemiology Synthesis Project database by the World Health Organization Regional Office for the Eastern Mediterranean. 2014.

45. Palestinian Health Information Center. Health Annual Report 2006. URL: <http://www.moh.ps/?lang=1&page=4&id=142:> 2007.

46. Palestinian Health Information Center. Health Annual Report 2003. URL: <http://www.moh.ps/?lang=1&page=4&id=137:> 2004.

47. National AIDS Programme and Central Statistics Bureau (of Syrian Ministry of Health). Knowledge, attitude, and professional behavior of health care workers around certain blood-borne diseases including AIDS and hepatitis B and C. Syrian Ministry of Health, United Nations Development Programme, and the World Health Organization, date unknown.

48. Othman BM, Monem FS. Prevalence of hepatitis C virus antibodies among health care workers in Damascus, Syria. Saudi medical journal. 2001;22(7):603-5. Epub 2001/08/02. PubMed PMID: 11479642.

49. Othman BM, Monem FS. Prevalence of hepatitis C virus antibodies among intravenous drug abusers and prostitutes in Damascus, Syria. Saudi medical journal. 2002;23(4):393-5. Epub 2002/04/16. PubMed PMID: 11953762.

50. al-Azmeh J, Frosner G, Darwish Z, Bashour H, Monem F. Hepatitis E in Damascus, Syria. Infection. 1999;27(3):221-3. Epub 1999/06/23. PubMed PMID: 10378137.
